# Skipper genome sheds light on unique phenotypic traits and phylogeny

**DOI:** 10.1186/s12864-015-1846-0

**Published:** 2015-08-27

**Authors:** Qian Cong, Dominika Borek, Zbyszek Otwinowski, Nick V. Grishin

**Affiliations:** Howard Hughes Medical Institute, University of Texas Southwestern Medical Center, 5323 Harry Hines Boulevard, Dallas, TX 75390-9050 USA; Department of Biophysics and Biochemistry, University of Texas Southwestern Medical Center, 5323 Harry Hines Boulevard, Dallas, TX 75390-8816 USA

**Keywords:** *Lerema accius*, Skipper butterflies, Whole genome, Comparative genomics, Lepidoptera, Genotype and phenotype, Phylogeny

## Abstract

**Background:**

Butterflies and moths are emerging as model organisms in genetics and evolutionary studies. The family Hesperiidae (skippers) was traditionally viewed as a sister to other butterflies based on its moth-like morphology and darting flight habits with fast wing beats. However, DNA studies suggest that the family Papilionidae (swallowtails) may be the sister to other butterflies including skippers. The moth-like features and the controversial position of skippers in Lepidoptera phylogeny make them valuable targets for comparative genomics.

**Results:**

We obtained the 310 Mb draft genome of the Clouded Skipper (*Lerema accius*) from a wild-caught specimen using a cost-effective strategy that overcomes the high (1.6 %) heterozygosity problem. Comparative analysis of *Lerema accius* and the highly heterozygous genome of *Papilio glaucus* revealed differences in patterns of SNP distribution, but similarities in functions of genes that are enriched in non-synonymous SNPs. Comparison of Lepidoptera genomes revealed possible molecular bases for unique traits of skippers: a duplication of electron transport chain components could result in efficient energy supply for their rapid flight; a diversified family of predicted cellulases might allow them to feed on cellulose-enriched grasses; an expansion of pheromone-binding proteins and enzymes for pheromone synthesis implies a more efficient mate-recognition system, which compensates for the lack of clear visual cues due to the similarities in wing colors and patterns of many species of skippers. Phylogenetic analysis of several Lepidoptera genomes suggested that the position of Hesperiidae remains uncertain as the tree topology varied depending on the evolutionary model.

**Conclusion:**

Completion of the first genome from the family Hesperiidae allowed comparative analyses with other Lepidoptera that revealed potential genetic bases for the unique phenotypic traits of skippers. This work lays the foundation for future experimental studies of skippers and provides a rich dataset for comparative genomics and phylogenetic studies of Lepidoptera.

**Electronic supplementary material:**

The online version of this article (doi:10.1186/s12864-015-1846-0) contains supplementary material, which is available to authorized users.

## Background

Butterflies and moths (Lepidoptera) have relatively small genomes compared to other eukaryotes, yet they display complex life cycles and diverse wing patterns. They are emerging as powerful models for genetic and evolutionary studies. A new paradigm that gene exchange between species being a driver in the evolution of adaptation in Heliconius butterflies, has increased excitement in the field [1]. Additional interest in the Lepidoptera models has resulted from discovering molecular mechanisms responsible for complex traits, such as sexual dimorphism [2–5].

Despite the wealth of life cycle, behavioral and morphological data available for butterflies (Rhopalocera), their phylogeny is uncertain. Traditionally, the Papilionidae (swallowtails), Pieridae, Nymphalidae, Lycaenidae and Riodinidae families were grouped into a single superfamily, Papilionoidea, which represents typical butterflies. A sister superfamily Hesperioidea contained a single family, Hesperiidae [6]. Hesperiidae are similar to many typical butterflies in the egg, larval and pupal stages, however, adults are morphologically distinct, and are characterized by reflexed antennal clubs, larger heads, and several moth-like characteristics such as stockier bodies, stronger wing muscles and darting flight with faster wing beats [6]. Their ability to fly rapidly gained them the common name “skippers”. Skippers were traditionally considered to be the basal branch of butterflies based on morphological characters [6]. Phylogenetic reconstructions of 57 butterfly and skipper species combining DNA sequences of three phylogenetic markers with morphological characters agreed with the basal placement of skippers [7]. However, a purely DNA-based phylogeny presented in the same study contradicted this view and placed Papilionidae at the base with Hesperiidae as a sister to other butterfly families. Similarly, a recent larger-scale study that included transcriptomes of 9 butterflies and skippers reported a highly confident (by bootstrap) phylogeny with Papilionidae in the basal position [8]. Therefore, reconciliation of the discrepancy between these morphology-based and DNA-based phylogenies requires further studies, and the phylogeny of major families of butterflies remains an open question.

**Fig. 1:**
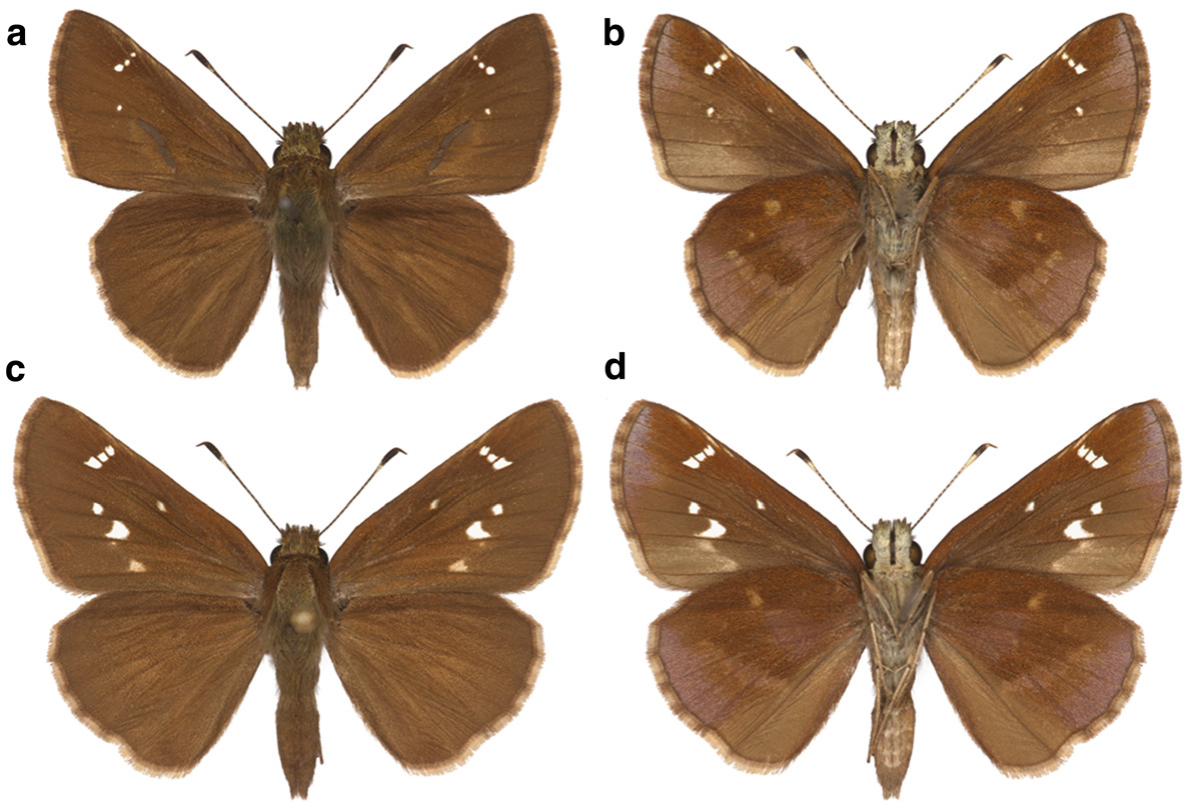
Photographs of *Lac* specimens. The specimens were reared from caterpillars collected near the Grapevine Lake (USA: Texas, Denton County, Flower Mound). **a** Dorsal and **b** ventral aspects of a male specimen, eclosed on 31-Jul-1997; **c** dorsal and **d** ventral aspects of a female specimen, eclosed on 29-Sep-1997

Decoding the skipper genomes could help the reconstruction of the Lepidoptera tree and provide information that is essential for understanding the evolution of their moth-like morphological features, which are either inherited from their ancestor or are character reversals. Here we report the assembly and gene annotations for the highly heterozygous genome of the Clouded Skipper, *Lerema accius* (J. E. Smith, 1797), abbreviated as *Lac*, shown on Fig. 1. *Lac* belongs to the subfamily Hesperiinae, commonly known as Grass Skippers, the most species-rich subfamily of skippers. Caterpillars of most Hesperiinae feed on grasses and sedges. Hesperiinae adults typically hold wings erect over the thorax and abdomen when feeding and resting. They adopt a “jet plane” pose when basking: partially open the wings and hold the fore- and hindwings at different angles.

Comparative analysis of this first genome from the family Hesperiidae with other Lepidoptera genomes provides hypotheses about genetic bases for unique morphological traits of skippers, such as their fast flight. Phylogenetic analyses of *Lac* and other Lepidoptera species with available complete genomes fail to resolve the position of Hesperiidae. A maximum likelihood tree constructed by RAxML [9] using the most suitable evolutionary model (JTTDCMUT model) selected by the program places swallowtails at the base of the tree, consistent with published DNA phylogenies, while Bayesian inference [10] with an evolutionary model that accounts for site-heterogeneity [11], supports the traditional morphology-based phylogeny in which skippers are the basal branch of butterflies. More extensive taxon sampling and/or more advanced methods of phylogenetic analysis are needed to resolve the position of Hesperiidae conclusively, and the first Hesperiidae genome provides a starting point for these studies.

## Results and discussion

### Genome quality assessment and gene annotation

We assembled a 310 Mb genome of *Lac* and compared its quality with genomes (Table 1, Additional file 1: Table S2A) of the following Lepidoptera species: *Plutella xylostella* (Pxy), *Bombyx mori* (*Bmo*), *Papilio glaucus* (*Pgl*), *Melitaea cinxia* (*Mci*), *Heliconius melpomene* (*Hme*), and *Danaus plexippus* (*Dpl*) [1–3, 12–17]. The scaffold N50 of *Lac* is 513 kb, which is longer than several other butterfly genomes. The genome is among the best in terms of completeness measured by the presence of CEGMA (Core Eukaryotic Genes Mapping Approach) genes [18], cytoplasmic ribosomal proteins and independently assembled transcripts. The residue coverage (86.6 %) of CEGMA genes (Additional file 1: Table S2B) by single *Lac* scaffolds is comparable to the residue coverage by the current *Bmo* assembly with an N50 of about 4.0 Mb, indicating that the quality of the *Lac* draft is sufficient for protein annotation and comparative analysis. This Whole Genome Shotgun project has been deposited at DDBJ/EMBL/GenBank under the accession LGAG00000000. The version described in this paper is version LGAG01000000. In addition, the main results from genome assembly, annotation and analysis can be downloaded at http://prodata.swmed.edu/LepDB/.

**Table 1:** Quality and composition of Lepidoptera genomes

Feature	*Lac*	*Pgl*	*Dpl*	*Hme*	*Mci*	*Bmo*	*Pxy*
Genome size (Mb)	310	376	249	274	390	480	394
Genome size without gap (Mb)	292	362	242	270	361	432	387
Heterozygosity (%)	1.6	2.3	0.55	n.a.	n.a.	n.a.	~2
Scaffold N50 (kb)	513	230	716	194	119	3999	737
CEGMA (%)	99.3	99.3	99.3	98.0	98.7	99.3	98.0
Average CEGMA coverage by single scaffold (%)	86.6	86.8	87.3	86.4	79.1	86.7	84.0
Cytoplasmic Ribosomal Proteins (%)	98.9	98.9	98.9	94.6	94.6	97.8	93.5
*De novo* assembled transcripts (%)	97 ~ 99	98	96	n.a.	~97	98	83
GC content (%)	34.4	35.4	31.6	32.8	32.6	37.7	38.3
Repeat (%)	15.5	22.0	16.3	24.9	28.0	44.1	34.0
Exon (%)	6.96	5.07	8.40	6.38	6.36	4.03	6.35
Intron (%)	31.6	25.6	28.1	25.4	30.7	15.9	30.7
Number of proteins (thousands)	17.4	15.7	15.1	12.8	16.7	14.3	18.1
Number of universal ortholog lost	153	114	47	354	521	394	1188
Number of species specific genes	4586	3172	2361	1526	4691	2486	5260

*n.a.* Data not available

We assembled the transcriptomes from two other *Lac* specimens, a pupa and an adult. Based on the transcriptomes, homologs from other insects, *de novo* gene predictions and repeat identification (Additional file 2: Table S3A), we predicted 17,416 protein-coding genes in *Lac* genome (Additional file 2: Table S3B). 79 % of these genes are likely expressed, as they fully or partially overlap with the transcripts. We annotated the putative function for 12,283 protein-coding genes and the annotations are listed in Additional file 2: Table S3C.

### Comparison of Lepidoptera genomes

**Fig. 2:**
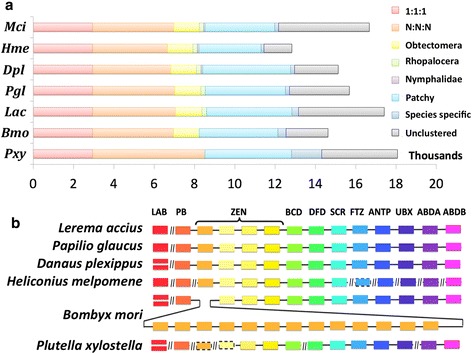
Comparative analysis of Lepidoptera genomes. **a** Number of different types of orthologs in each Lepidoptera species with published genomes. 1:1:1: single-copy orthologs shared among all species; N:N:N: multiple-copy orthologs shared among all species, i.e. more than one copy in at least one species; Obtectomera: orthologs specific to Obtectomera, i.e. all other six species except *Pxy*; Rhopalocera: orthologs specific to Rhopalocera, i.e. all other five species except *Bmo* and *Pxy*; Nymphalidae: orthologs specific to Nymphalidae, i.e. *Dpl*, *Mci* and *Hme*; Patchy: orthologs that are shared between more than one, but not all species (excluding those belongs to previous categories); Species-specific: specific to only one species and having close homologs within that species; Unclustered: proteins that do not belong to any of the orthologous groups. **b** Arrangements of Hox genes in Lepidoptera genomes. Orthologs are shown as boxes of the same color; double boxes in the same position indicate gene duplications, dashed-line around a box implies that this gene is missing in the genome assembly but present in the transcriptome; “//” marks the boundaries between different scaffolds

We compared the composition of the *Lac* genome with that of other Lepidoptera (Table 1). Although the genome sizes of Lepidoptera range from 250 to 500 Mbp, the total lengths of coding regions are comparable. The reported repeat content of these genomes varies significantly, and it is positively correlated with the genome size. We identified orthologous proteins encoded by these genomes and detected 5770 universal orthologous groups and 2940 of them consist of a single-copy gene in each of the species (Fig. 2a). We compared two protein families: Hox genes that are crucial for development and Odorant Receptors (OR) that are particularly important for the feeding and mating behaviors of insects. *Lac* had the same set of Hox genes as other Lepidoptera (Additional file 3: Table S4A). All the *Lac* Hox genes that are expected to be linked are located on the same scaffold in the order typical for Lepidoptera (Fig. 2b). The *Lac* genome encodes 56 ORs, which is comparable to *Pgl* but less than *Hme*, *Dpl* and moths (Additional file 3: Table S4B). The *Mci* genome appears to encode the smallest number of ORs (48), but this number is likely underestimated because of the poor continuity of the current *Mci* genome assembly (119 kbp). Clustering analysis (Additional file 4: Figure S2) shows that ORs in Lepidoptera can be classified into several subfamilies, and the *Lac* genome encodes ORs from each of these subfamilies.

### Functional implication of non-random single-nucleotide polymorphism (SNP) distribution

**Fig. 3:**
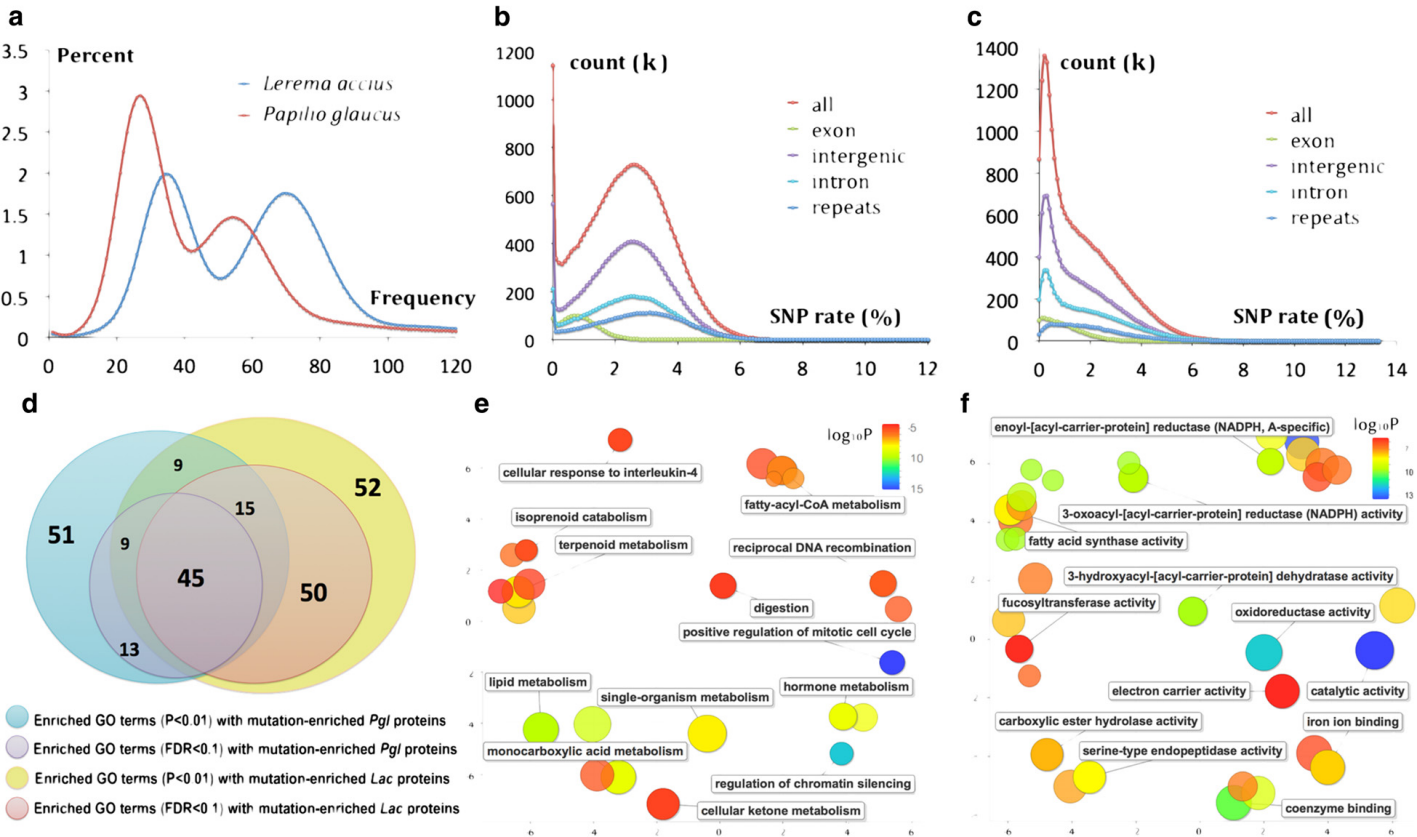
Analysis of SNPs in the *Lac* and *Pgl* genomes. **a** Histograms of 17-mer frequency in the sequence reads of *Lac* (*blue*) and *Pgl* (*red*). For both species, the peak on the left represents frequency distribution of 17-mers from heterozygous regions and the peak on the right is for homozygous regions. The relative heights of the two peaks is an indicator for heterozygosity level. **b** Histogram of SNP rates in 1000 bp overlapping windows from different regions in the *Pgl* genome. **c** Histogram of SNP rates in 1000 bp overlapping windows from different regions in the *Lac* genome. **d** Venn diagram showing the large overlap between enriched GO terms associated with mutation-enriched proteins in both *Lac* and *Pgl* genomes. **e** Enriched GO terms (in the category of biological processes) associated substitution-enriched proteins in both *Pgl* and *Lac*. GO terms are grouped in space by similarity in meaning and colored by the level of significance (scale shown in the upper left corner), which is a product of p-values for this GO term’s enrichment in *Pgl* and *Lac* genomes. Annotations are shown for the representative GO-terms for groups of similar terms. **f** Similar to (**e**), but these GO terms belong to the category of molecular function

The *Lac* genome is highly heterozygous, as suggested by the distribution of k-mer frequencies (Fig. 3a). We compared heterozygosity properties of the *Lac* genome with the highly heterozygous *Pgl* genome that we previously assembled [16]. Approximately 2.3 % of the positions in the *Pgl* genome and 1.6 % of the positions in the *Lac* genome are different between the two homologous chromosomes. In both genomes, the SNP rate in the coding regions (0.91 % for *Pgl* and 0.96 % for *Lac*) is much lower than that for the non-coding regions (2.4 % for *Pgl* and 1.7 % for *Lac*) (Additional file 4: Figure S3a), which is likely due to the potential deleterious effect of SNPs in the coding regions.

Both the *Pgl* and *Lac* genomes contain long segments (>1,000) that are free of SNPs. However, the SNP-free segments in the *Pgl* genome are significantly longer than those in *Lac*. The longest SNP-free segments in *Pgl* and *Lac* are about 734.8 kbp and 13.5 kbp, respectively. One possible explanation for the presence of SNP-free regions is that their high heterozygosity prevents the mapping of reads from the alternative homologous chromosomes, resulting in failures to detect SNPs. But this explanation is not likely the dominant reason, since we included only regions with coverage that is expected for a diploid genome in this analysis (Additional file 4: Figure S3b,c). Another potential cause for SNP-free regions is that insects frequently inbreed in nature and that the parents of the sequenced specimen could share a recent common ancestor, from which they inherited the same alleles.

Omitting the SNP-free regions, the distribution of SNP rates in the *Pgl* genome can be approximated by a single normal distribution (Fig. 3b and Additional file 4: Figure S3d). In contrast, the distribution of SNPs rates (Fig. 3c and Additional file 4: Figure S3e) in the *Lac* genome can be represented by a mixture of two Gaussian distributions: one centered around 0.3–0.4 % and a second centered at 2.5 %. We speculate that a SNP rate of 0.3–0.4 % corresponds to the variation accumulated within the local population of *Lac*, whereas the higher SNP rates in certain regions reflect gene flow from other populations or even from other species. Human activities might have an impact on the high SNP rates of *Lac*. *Lac* feeds on widely planted grasses (*Poaceae* family). Expansion of this common food source by humans might cause previously isolated *Lac* populations to meet.

About a quarter (22 % for *Lac* and 26 % for *Pgl*) of the SNPs in the protein coding regions are non-synonymous and result in amino acid substitutions in proteins. Segments in proteins that are predicted to be structurally disordered are significantly more enriched in substitutions (Additional file 4: Figure S3f). This enrichment is likely due to higher tolerance of disordered regions to substitutions [19]. To understand the functional consequence of SNPs in the *Pgl* and *Lac* genomes, we identified proteins that are significantly enriched (false discovery rate < 0.1) in substitutions in their structurally ordered regions (Additional file 5: Table S5A, B).

The enriched GO terms (Additional file 5: Table S5C, D) associated with substitution-enriched proteins in both genomes show a significant (*p* < 1e-15) overlap (Fig. 3d). Among the enriched biological processes (Fig. 3e) and molecular functions (Fig. 3f) in both species, the molecular function “catalytic activity” is among the most significant (*p* < 1e-4). Approximately 40 % of the substitution-enriched proteins are enzymes in both species. The most significantly enriched GO (*p* < 1e-8) terms for *Lac* (GO:0045931, GO:0031935, GO:0060968, GO:0045787 and GO:0030178) can each be attributed solely to a single substitution-rich protein family: C2H2 zinc fingers. Both insect and mammalian genomes encode large numbers of C2H2 zinc fingers, and their exact function is not fully understood [20]. However, C2H2 zinc fingers were implicated to function in transcriptional silencing of exogenous DNA [21, 22]. We hypothesize that the C2H2 zinc fingers evolved adaptively as the population was exposed to exogenous DNA sources, such as retrovirus or gene flow from other species.

### Phylogenetic analysis with whole-genome data

The morphology-based view of butterfly evolution suggests a tree topology (((((*Mci*, *Hme*), *Dpl*), *Pgl*), *Lac*), *Bmo*, *Pxy*) [6, 7], whereas recent DNA-based phylogenetic analyses support an alternative topology (((((*Mci*, *Hme*), *Dpl*), *Lac*), *Pgl*), *Bmo*, *Pxy*) [7, 8]. We refer to these two topologies as the traditional topology and the alternate topology, respectively.

**Fig. 4:**
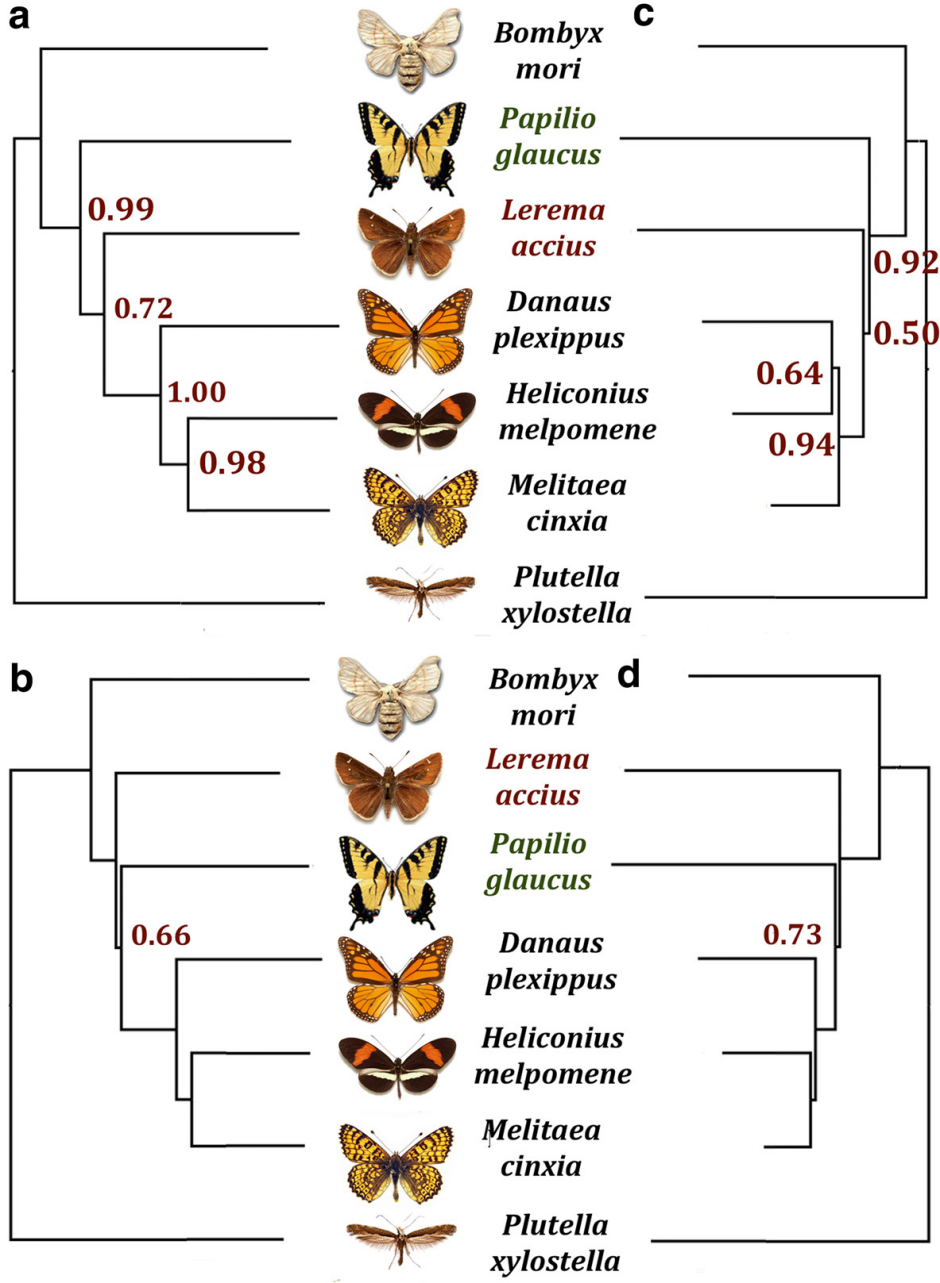
Phylogenetic trees of the butterflies based on whole-genome data. **a** Majority-rule consensus tree of the maximal likelihood trees constructed by RAxML on 1000 random samples from the concatenated alignment of universal single-copy orthologs. **b** Neighbor-joining tree based on the frequency of gene rearrangement events between species. **c** Consensus tree of the better-supported trees inferred from PhyloBayes analyses on 1000 random samples from the concatenated alignment of universal single-copy orthologs. The tree topology was constrained to either of the two reported topologies: (((((*Mci*, *Hme*), *Dpl*), *Lac*), *Pgl*), *Bmo*, *Pxy*) or (((((*Mci*, *Hme*), *Dpl*), *Pgl*), *Lac*), *Bmo*, *Pxy*). **d** Phylogenetic tree using the gene-rearrangement data inferred by PhyloBayes with CAT model

Whole-genome sequences of these species allowed us to model their phylogeny using both the alignments of universal single-copy orthologs and the synteny of genes. However, both the traditional and the alternate tree topologies can be supported by the data depending on which evolutionary models and tree construction methods are selected. The 50 % majority rule consensus tree of maximum likelihood trees constructed with RAxML [9] on the alignments of individual proteins failed to completely resolve the phylogeny (Additional file 4: Figure S4) due to short lengths (median length: 209 amino acids) of individual alignments. Instead, a similar consensus tree built on 1000 random samples (>5,000 aligned positions) from the concatenated alignment agreed with the alternate topology (Fig. 4a). However, the clade that groups skippers with other butterflies is only the best solution in 72 % of random samples.

To further test which topology is better supported, we used the Bayesian phylogenetic analysis software PhyloBayes [10] with the CAT model [11] that accounts for site heterogeneity in amino acid substitutions by dividing the sites into 4 categories. We constrained the tree topology to either the traditional or the alternative one. This analysis supported the traditional topology in 66 % of the 1000 random samples. A consensus tree summarizing the tree topologies with higher likelihood based on each data set is shown in Fig. 4b.

Similarly, the phylogeny inferred from gene rearrangement events (Additional file 3: Table S4C) produced different results depending on the selection of evolutionary model. While a simple neighbor-joining tree based on the frequency of gene arrangement supported the alternative topology (Fig. 4c), Bayesian interference with the CAT model supported the traditional topology with a higher likelihood (Fig. 4d). Our analyses suggest that the traditional tree topology based on morphological features is not contradicted by the genomic data, but the uncertainty of reconstructions is too high to conclusively determine the evolutionary history of butterflies.

The discrepancy between morphological and molecular phylogeny has been a long-standing problem in evolutionary biology [23]. The incongruence between molecular trees obtained with different methods or different data sets is also frequently encountered [23, 24] Studies on several other systems reveal similar uncertainty as we observed in our analysis [25, 26]. This uncertainty in butterfly phylogeny may also result from incomplete lineage sorting [27]. Trees built from different orthologous groups support different topologies with high bootstrap values. Out of the 522 maximum likelihood trees of individual orthologous groups with bootstrap support above 80 %, a significant portion supports the traditional topology (24.7 %) and a third possible topology (33.7 %) that groups *Pgl* and *Lac* in a clade. In addition, the limited number of available butterfly genomes impedes a better taxon sampling for the phylogenetic reconstruction of butterflies. Genome sequences of species that represent the early branches in each family of butterflies could help to resolve the uncertainty in the phylogenetic tree of butterflies.

### Expanded gene families in *Lac* suggest possible genetic bases for phenotypic traits

**Fig. 5:**
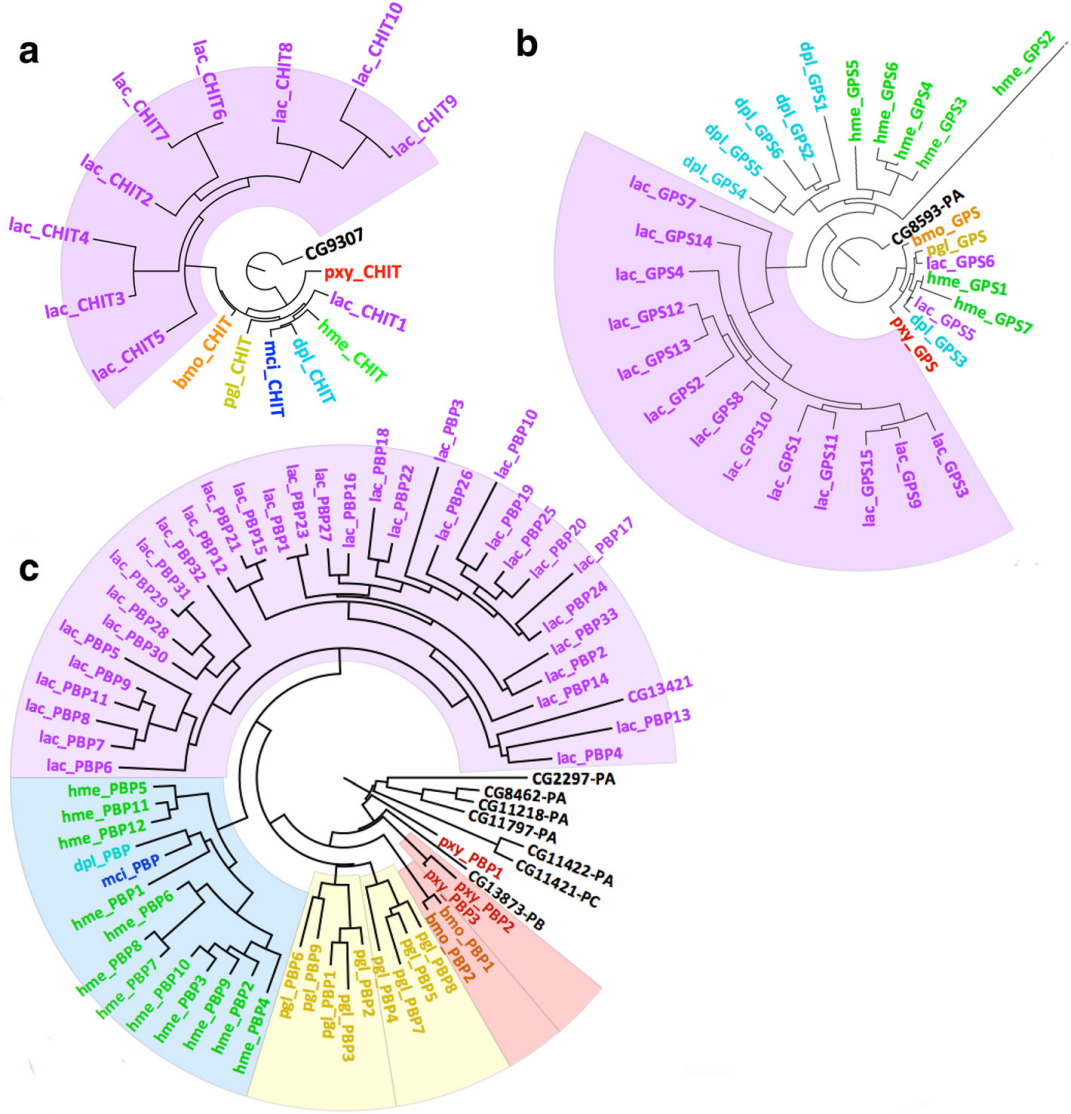
Phylogenetic trees for expanded protein families in *Lac.* Abbreviation of the species and protein names are used as labels in the phylogenetic trees. We colored the labels to indicate which species the protein is from: *Lac* (*purple*), *Pgl* (*dark yellow*) *Dpl* (*cyan*), *Hme* (*green*), *Mci* (*blue*), *Bmo* (*orange*), *Pxy* (*red*) and *Drosophila melanogaster* (*black*). The clades corresponding to the unique gene expansion events in *Lac* are highlighted in light magenta. **a** Phylogenetic tree of endochitinases. **b** Phylogenetic tree of Geranylgeranyl pyrophosphate synthases. **c** Phylogenetic tree of Pheromone-binding proteins.

Compared to other Lepidoptera species, the *Lac* genome expands in several protein families (Additional file 6: Table S6). Endochitinase-like proteins are uniquely expanded (Fig. 5a) and cluster on the same scaffold in the genome, which indicates that they originated from recent gene duplication events. As shown in the phylogenetic tree (Fig. 5a), these duplicated endochitinase-like proteins diverged rapidly and only one copy retained high sequence similarity to the orthologous proteins in other Lepidoptera and *Drosophila melanogaster* genomes. While this single conserved copy likely preserves the function of endochitinase, we hypothesize that the other divergent endochitinase-like proteins could have adopted new functions to digest cellulose. This hypothesis is based on the following four facts: (1) *Lac* and most skippers in the Hesperiinae subfamily feed on the cellulose-rich grasses; (2) the *Lac* genome and other Lepidoptera genomes do not encode proteins that belong to the families of known cellulases; (3) endochitinases are homologs of cellulases and they are structurally very similar (Additional file 4: Figure S5) [28]; (4) cellulose and chitin are structurally similar and they are both digested through glycoside hydrolysis. Therefore, these endochitinase-like proteins in *Lac* may have evolved to digest cellulose, allowing *Lac*, and possibly other grass-feeding skippers in the Hesperiinae subfamily, to feed on grasses that are rich in cellulose. It remains to be explored if other Monocot feeders, such as Satyrinae (Nymphalidae), have a similar expansion or use different enzymes.

Another expanded protein family is geranylgeranyl pyrophosphate synthase (GGPPS, Fig. 5b) homologs. GGPPSs are used in the biosynthesis of terpenes and terpenoids, which are frequently used as an intermediate products for pheromone biosynthesis. 13 copies of GGPPS homologs in *Lac* form a clade in the phylogenetic tree (highlighted in magenta in Fig. 5b) and their sequences have diverged from the Drosophila GGPPS. It is possible that these homologs have adopted slightly different functions and gained the ability either to catalyze different steps to synthesize one type of pheromone or to produce a wide range of different pheromone molecules. In addition, *Lac* encodes a much larger number of pheromone-binding proteins (PBPs) than other Lepidoptera species and these PBPs form a clade in the phylogenetic tree of Lepidoptera PBPs (Fig. 5c). Both gene expansion events suggest a more advanced pheromone production and sensing system in *Lac*. Butterflies can select their mates both by visual cues and by sensing pheromones at close range. However, many skipper species have similar wing colors and patterns, which might confuse recognition of mates from the same species by visual cues. Therefore, a stronger pheromone system in *Lac* might allow individuals to efficiently detect mates of the same species.

The phylogenetic tree of GGPPS homologs reveals two copies in *Lac* (annotated as *Lac*_GPS5 and *Lac*_GPS6) that clustered closely to the *Drosophila* GGPPS, rather than in the clade of other divergent GGPPS homologs. We speculate that these two copies are orthologs of the *Drosophila* GGPPS and retain similar function. *Drosophila* GGPPS was shown to be crucial for heart formation. It works in the mevalonate pathway and directly synthesizes GGPP, which can be transferred to G protein Gγ1. The geranylgeranylation of Gγ1 is required for heart formation [29]. The duplication of GGPPS may be related to heart development for efficient energy supply to sustain the rapid wing beats of *Lac*. In addition, several mitochondria targeted genes encoded by the nuclear genome are also duplicated in the *Lac* genome (Table 2), including components of the NADH dehydrogenase [uniquinone] complex, which are directly linked to energy production. The *Lac* genome is significantly (*p* < 1e-7) enriched in mitochondria targeted genes compared to other Lepidoptera as reflected by the GO terms. Taken together, we propose that the observed enrichment and duplications of mitochondrial proteins allow for dynamic adaptation of mitochondrial functions depending on type of organ, tissue, or life stage and ensure efficient energy supply for rapid wing beats in adults of *Lac*.

**Table 2:** Mitochondria-targeted proteins that are duplicated in *Lerema accius*

*Lerema accius* proteins	Function
lac1604.25, lac1604.24, lac947.51	NADH dehydrogenase [ubiquinone] 1 α subcomplex subunit 6
lac3140.17, lac2615.10, lac570.16	NADH dehydrogenase [ubiquinone] 1 α subcomplex subunit 11
lac279.21, lac34153.1	Heat shock protein 75 kDa
lac151.15, lac151.16	Acetyl-CoA acetyltransferase A
lac492.55, lac676.35	28S ribosomal protein S18b
lac5129.10, lac6133.18	2-oxoisovalerate dehydrogenase subunit β

## Conclusions

We report the draft genome of Clouded Skipper. Being the first sequenced genome from the Hesperiidae family, it offers rich data for comparative genomics and phylogenetic studies of Lepidoptera. We devised a cost-efficient protocol that overcomes the difficulty in assembling highly heterozygous genome. Despite the high level of heterozygosity (1.6 %), the quality of our genome assembly is nearly the best among published Lepidoptera genomes. This protocol should stimulate and enable sequencing of other insect genomes. Comparative analyses of Lepidoptera genomes suggest possible genetic bases for the unique phenotypic traits of skippers, including fast flight with rapid wing beats, ability to feed on grasses in larval stage, and recognize mates efficiently in spite of the similarity in wing patters of many species. These new data should facilitate experimental studies of skippers and contribute to the understanding of how diverse phenotypes are encoded by the genomes.

## Methods

### Library preparation and sequencing

We removed and preserved the wings and abdomen of a freshly caught and frozen male *Lac* specimen (USA: Texas: Dallas County, Dallas, White Rock Lake, Olive Shapiro Park, 10-Nov-2013, GPS: 32.8621, −96.7305, elevation: 141 m, specimen NVG-1769. The specimen will be deposited in the National Museum of Natural History, Smithsonian Institution, Washington, DC, USA (USNM)), and extracted approximately 15 μg genomic DNA from the rest of its body with the ChargeSwitch gDNA mini tissue kit. 250 and 500 bp paired-end libraries were prepared using enzymes from NEBNext Modules and following Illumina TruSeq DNA sample preparation guide. Three mate pair libraries (2kb, 6kb and 15kb) were prepared using a protocol that was modified from a previously published Cre-Lox-based method [30]. For the 250, 500, 2k, 6k and 15k bp libraries, approximately 500, 500, 1.5, 3 and 6 μg of DNA were used, respectively. A *Lac* adult and a pupa reared from a caterpillar collected at the same locality (White Rock Lake) were preserved in *RNAlater* solution and total RNA was extracted from them using QIAGEN RNeasy Plus Mini Kit. We further isolated mRNA using NEBNext Poly(A) mRNA Magnetic Isolation Module and RNA-seq libraries for both specimens were prepared with NEBNext Ultra Directional RNA Library Prep Kit for Illumina following manufactory’s protocol.

We quantified the amount of DNA from all the libraries with the KAPA Library Quantification Kit, and mixed 250, 500, 2k, 6k, 15k bp genomic DNA, pupal RNA-seq and adult RNA-seq libraries at  relative molar concentration 40:20:8:4:3:20:10 to get the final library. The final library was sent to the genomics core facility at UT Southwestern Medical Center for 150 bp paired-end sequencing on Illumina HiSeq2000. The sequencing reads to assemble the genome have been deposited in NCBI SRA database under accession numbers: SRR2089769- SRR2089775. The sequencing reads to assemble the transcriptomes have been deposited at the same database under accession numbers: SRR2089776 and SRR2089777.

### Genome assembly

We removed sequencing reads that did not pass the Illumina purity filter and classified the remainder according to their TruSeq adapter indices. Mate pair libraries were processed by the Delox script [30] to remove the LoxP sequences and to separate true mate pair from paired-end reads. All reads were processed by mirabait [31] to remove contamination from the TruSeq adapters, fastq_quality_trimmer (from FASTX_TOOLKIT, http://hannonlab.cshl.edu/fastx_toolkit/) to remove low quality portions at both ends, JELLYFISH [32] to obtain k-mer frequencies in all the libraries, and QUAKE [33] to correct sequencing errors (Additional file 7: Table S1). The data processing resulted in nine libraries that were supplied to Platanus [34] for genome assembly: 250 and 500 bp paired-end libraries, three paired-end and three mate pair libraries from 2, 6 to 15 kb libraries and a single-end library containing all reads whose pairs were removed in the process.

We mapped these reads to the initial assembly with Bowtie2 [35] and calculated the coverage of each scaffold with the help of SAMtools [36]. Many short scaffolds in the assembly showed coverage that was about half of the expected value, which likely resulted from highly heterozygous regions that were not merged to the equivalent segments in the homologous chromosomes. We merged them into other scaffolds if they could be fully aligned (coverage > 90 % and uncovered region < 500 bp) to another significantly less covered region in a longer scaffold with high sequence identity (>95 %). Similar problems occurred in the *Heliconius melpomene* and *Papilio glaucus* genome projects, and similar strategies were used to improve the assemblies [1, 16].

### Transcriptome assembly

After removing contamination from TruSeq adapters and the low quality portion of the reads using the methods mentioned above, we applied three methods to assemble the transcriptomes: (1) *de novo* assembly by Trinity [37], (2) reference-based assembly by TopHat [38] (v2.0.10) and Cufflinks [39] (v2.2.1), and (3) reference-guided assembly by Trinity. The results from all three methods were then integrated by Program to Assemble Spliced Alignment (PASA) [40].

### Identification of repeats and gene annotation

Two approaches were used to identify repeats in *Lac* genome: the RepeatModeler [41] pipeline and in-house scripts that extracted regions with coverage 4 times higher than expected. These repeats were submitted to the CENSOR [42] server to assign them to the repeat classification hierarchy. The species-specific repeat library and repeats classified in RepBase [43] (V18.12) were used to detect repeats in the genome by RepeatMasker [44].

From the transcripts of both specimens in the pupal and adult stages, we obtained two sets of transcript-based annotations from two pipelines: TopHat followed by Cufflinks and Trinity followed by PASA. In addition, we obtained five sets of homology-based annotations by aligning protein sets from *Drosophila melanogaster* [45] and four published Lepidoptera genomes to the *Lac* genome with exonerate [46]. Proteins from the entire UniRef90 [47] database were used to generate another set of gene predictions by genblastG [48]. We manually curated and selected 1427 confident gene models by integrating the evidence from transcripts and homologs to train *de novo* gene predictors: AUGUSTUS [49], SNAP [50] and GlimmerHMM [51]. These trained predictors, the self-trained Genemark [52] and a consensus based pipeline, Maker [53] were used to generate another five sets of gene models. Transcript-based and homology-based annotations were supplied to AUGUSTUS, SNAP and Maker to boost their performance. In total, we generated 15 sets of gene predictions and integrated them with EvidenceModeller [40] to generate the final gene models.

We predicted the function of *Lac* proteins by transferring annotations and GO-terms from the closest BLAST [54] hits (E-value < 10^−5^) in both the Swissprot [55] database and Flybase [56]. Finally, we performed InterproScan [57] to identify conserved protein domains and functional motifs, to predict coiled coils, transmembrane helices and signal peptides, to detect homologous 3D structures, to assign *Lac* proteins to protein families and to map them to metabolic pathways.

### Assembly quality assessment and comparison to other Lepidoptera genomes

We obtained the most recent versions of other published Lepidoptera genomes, including *Bombyx mori*, *Danaus plexippus*, *Heliconius melpomene*, *Melitaea cinxia*, *Papilio glaucus*, and *Plutella xylostella* [1–3, 12–17]. Using the criteria applied in the Monarch butterfly genome paper [2], we estimated the completeness of these genomes based on their coverage of independently obtained transcripts, CEGMA [18] genes and the Cytoplasmic Ribosomal Proteins.

We compared various properties of these published genomes and clustered the proteins annotated in them using OrthoMCL [58]. We identified the Hox genes using homeodomains from *Drosophila* in the HomeoDB [59] as reference, and relationship among them were detected using a phylogenetic tree built by RAxML [9] with automatically selected model on the MAFFT [60] alignment. Starting from the annotated odorant receptors from the *Bmo*, *Hme* and *Dpl* genomes, we identified all the odorant receptors in the annotated protein sets from these Lepidoptera genomes using reciprocal BLAST. Odorant receptors encoded by the genome but missed in the protein sets were predicted with the help of genblastG. All the candidates identified by the automatic programs were further curated to remove short fragments (<200 aa) and false positive hits that do not detect odorant receptors as the top hit in a BLAST search against Flybase entries. Sequences of these odorant receptors were compared and clustered using CLANS [61].

### Detection and analysis of SNPs

We analyzed the SNPs in *Lac* and *Pgl* genomes using the same protocol, in which we mapped the sequence reads to the genomes and detected SNPs using the Genome Analysis Toolkit [62]. The distribution of genome coverage by the reads in 100 bp windows was plotted in Additional file 4: Figure S3b,c. For both *Pgl* and *Lac* genomes, this distribution shows two peaks. In addition to the main peak centered at the expected coverage for a diploid genome, there is an additional peak to the left that corresponds to highly divergent regions between the two homologous chromosomes. Owing to this sequence divergence, only the reads corresponding to the sequence of one of the homologous chromosomes can be mapped, which results in the lower-than-expected coverage. To analyze the distribution of SNPs, we focused on the regions, in which coverage by the reads falls within the diploid peak. We divided these regions into exons, introns, repeats and intergenic regions. The percent of SNPs in overlapping 1000 bp windows in the genome was used to reflect their distributions. We detected non-synonymous SNPs that will cause substitutions in proteins and predicted structurally disordered regions in proteins with ESpritz server [63].

We identified proteins with significantly more substitutions with binomial tests (p = average percent of substitutions in all proteins, m = number of substitutions in a protein, N = length of a protein) followed by False Discovery Rate (FDR) tests [64]. We considered proteins with Q-values (maximal FDR level) smaller than 0.1 to be significantly enriched in substitutions. We excluded the regions that were predicted to be structurally disordered and performed similar tests. Enriched GO terms associated with these substitution-enriched proteins were identified with another binomial test (P = probability of this GO-term being associated with any protein, m = number of substitution-enriched proteins associated with this GO-term, N = number of substitution-enriched proteins). The significantly enriched GO terms were submitted to the REVIGO [65] web server to cluster similar GO terms and visualize them.

### Phylogenetic tree reconstruction

We performed the phylogenetic analysis based on the 2940 universal single-copy orthologs in the Lepidoptera genomes (*Lac*, *Bmo*, *Pxy*, *Dpl*, *Hme*, *Mci*, and *Pgl*) detected by OrthoMCL. We built alignment for each orthologous group using both global sequence aligner MAFFT and local sequence aligner BLASTP. 570,686 positions that were consistently aligned by both aligners were extracted. All the alignments were concatenated and the aligned positions were randomly divided to 100 groups, so that each group contained about 5,706 or 5,707 aligned positions. We repeated this procedure 10 times to obtain a total of 1,000 representative alignments for phylogenetic analysis. In addition, the 1,991 alignments of individual orthologous groups containing more than 100 aligned positions were used as a separate data set in the phylogenetic analysis.

We used two methods for phylogenetic analysis: a maximum likelihood method RAxML, in which the evolutionary model is automatically selected by the program based on the data and a Bayesian inference method PhyloBayes [10] with CAT model that divide sites into categories and account for site-heterogeneities [11]. In addition to allowing the program to search for the best tree topologies, we constrained the Bayesian analysis to two previously observed topologies: (((((*Mci*, *Hme*), *Dpl*), *Lac*), *Pgl*), *Bmo*, *Pxy*) and (((((*Mci*, *Hme*), *Dpl*), *Pgl*), *Lac*), *Bmo*, *Pxy*). We compared the posterior probabilities given the two topologies imposed as priors to select the tree topology that is better supported by the data.

In addition, we used the frequencies of gene rearrangements to construct phylogenetic trees. As illustrated in Additional file 4: Figure S4, we started from the 5770 orthologous families present in each of the 7 species and removed families with extensive gene duplications (more than 4 copies of a gene in any species), which resulted in 5639 families. In each species, we determined the relative genomic orientation for every pair of gene families on the same scaffold. There are four possible relative orientations: [a+, b+]; [a-, b-]; [a+, b-]; [a-, b+], where a and b are genes from two families and “+” and “-” indicate the DNA strand they are encoded on. Due to the limited continuity of draft genomes, relative orientations in all 7 species could be determined for only 2120 such gene pairs. Then, we restricted the analysis to 1121 pairs so that each family participated in only one pair. We used four letters (A, B, C, and D) to denote the relative orientations of family pairs, and expressed the arrangement of the 1121 pairs in each species by a string of these letters. These strings were used as input for PhyloBayes for tree construction. The numbers of differences between these strings were used as evolutionary distances between species to construct phylogenetic tree with BioNJ [66].

### Analysis of gene expansion in *Lac*

We identified the closest homolog (BLASTP e-value < 0.00001) of each Lepidoptera protein in Flybase. If two OrthoMCL-defined orthologous families in Lepidoptera shared a common Flybase entry as their closest homolog, we merged them into one family. We considered *Lac* to have undergone gene expansion in a family if both the number and the total length of *Lac* proteins in this family are more than 1.5 times of the average number and total length for other Lepidoptera species. The most significantly expanded gene families with well-defined functions were further investigated using reciprocal BLAST results and function annotations to include all relevant proteins. Proteins encoded by the genomes but missed in the protein sets were predicted with the help of genblastG. Protein sequences from each family were aligned with MAFFT. Evolutionary trees were built with RAxML and visualized in FigTree.

## Availability of supporting data

See the Supplemental Information for detailed protocols. Major in-house scripts and intermediate results are available at http://prodata.swmed.edu/LepDB/.

## Additional files

Additional file 1: Table S2. Quality and composition of Lepidoptera genomes, related to Table 1. (XLSX 53 kb) Additional file 2: Table S3. Genome annotation. (XLSX 7627 kb) Additional file 3: Table S4. Comparative analyses of the Lepidoptera genomes, related to Figs. 2 and 4. (XLSX 113 kb) Additional file 4:
**Index for supplemental materials.** Supplemental Figures and Figure Legends. Extended Experimental Procedures. Supplemental Reference. (PDF 3832 kb) Additional file 5: Table S5. Distribution of SNPs in the *Papilio glaucus* and *Lerema accius* genomes, related to Fig. 3. (XLSX 226 kb) Additional file 6: Table S6. Gene expansion events in *Lerema accius*, related to Fig. 5. (XLSX 51 kb) Additional file 7: Table S1. Statistics for sequencing data, data processing and genome assembly, related to experimental procedure. (XLSX 15 kb)

## Abbreviations

*Lac*: *Lerema accius*
*Pgl*: *Papilio glaucus*
*Dpl*: *Danaus plexippus*
*Hme*: *Lerema accius*
*Mci*: *Melitaea cinxia*
*Bmo*: *Bombyx mori*
*Pxy*: *Plutella xylostella*
OR: Odorant receptors
FDR: False discovery rate
