# Supplementary material for: Skipper genome sheds light on unique phenotypic traits and phylogeny
Source: BMC Genomics. 2015 Aug 27;16(1):639. doi: 10.1186/s12864-015-1846-0 (PMC4551732; doi:10.1186/s12864-015-1846-0)
Supplement: Additional file 4: — Index for supplemental materials. Supplemental Figures and Figure Legends. Extended Experimental Procedures. Supplemental Reference. (PDF 3832 kb) [file 12864_2015_1846_MOESM4_ESM.pdf]

Skipper genome sheds light on unique phenotypic traits and phylogeny

Qian Cong[2], Dominika Borek[2], Zbyszek Otwinowski[2], and Nick V. Grishin[1,2]

[1]Howard Hughes Medical Institute, University of Texas Southwestern Medical Center, 5323 Harry Hines Boulevard, Dallas, Texas 75390-9050, USA. [2]Department of Biophysics and Biochemistry, University of Texas Southwestern Medical Center, 5323 Harry Hines Boulevard, Dallas, Texas 75390-8816, USA.

Index for supplemental materials

Supplemental Figures and Figure Legends **1-5**

Extended Experimental Procedures **6-47**

S1 Sequencing library preparation protocol **6-17**
S1.1 Genomic DNA extraction 6
S1.2 Total DNA extraction 7
S1.3 Paired-end library preparation protocol 8-9
S1.4 Mate pair library preparation protocol 10-14
S1.5 RNA-seq library preparation protocol 14-16
S1.6 Preparation for sequencing on the Illumina HiSeq platform 17

S2 Genome assembly strategy **18-20**
S2.1 Data processing and error correction 18-19
S2.2 Genome assembly 19
S2.3 Post-assembly improvement 19-20

S3 Transcriptome assembly strategy **21**
S3.1 Data processing 21
S3.2 *De novo* assembly, reference-guided assembly and mapping to the genome 21

S4 Genome assembly quality assessment **22-23**
S4.1 Genome assembly quality assessment by the coverage of transcripts 22
S4.2 Genome assembly quality assessment by the coverage of CEGMA genes 22-23
S4.3 Genome assembly quality assessment by Cytoplasmic Ribosomal Proteins 23

S5 Detection of SNPs in the genome **24-25**
S5.1 SNP detection 24
S5.2 Distribution of SNPs in different genomic regions 24
S5.3 Proteins enriched in substitutions 25

S6 Identification and classification of repeats **26**
S6.1 Construction of a species-specific repeat library 26
S6.2 Detection and masking of repeats in the genome 26

S7 Gene annotation **27-30**
S7.1 Transcript-based gene annotation 27
S7.2 Homology-based gene annotation 27
S7.3 *De novo* gene annotation 27-29
S7.4 Consensus-based final gene annotation 29-30
S7.5 Prediction of protein function and additional features 30

S8 Comparison of Lepidoptera genomes **31-33**
S8.1 Identification of orthologs 31
S8.2 Analysis of Hox genes 31
S8.3 Identification of Odorant Receptors (OR) 32
S8.4 Identification of expanded gene families 32
S8.5 In-depth study of important gene expansion events 33

S9 Phylogenetic analysis **34-35**
S9.1 Phylogenetic analysis based on universal single-copy orthologs 34
S9.2 Phylogenetic analysis based on gene re-arrangement events 34-35

Supplemental Reference 36-40

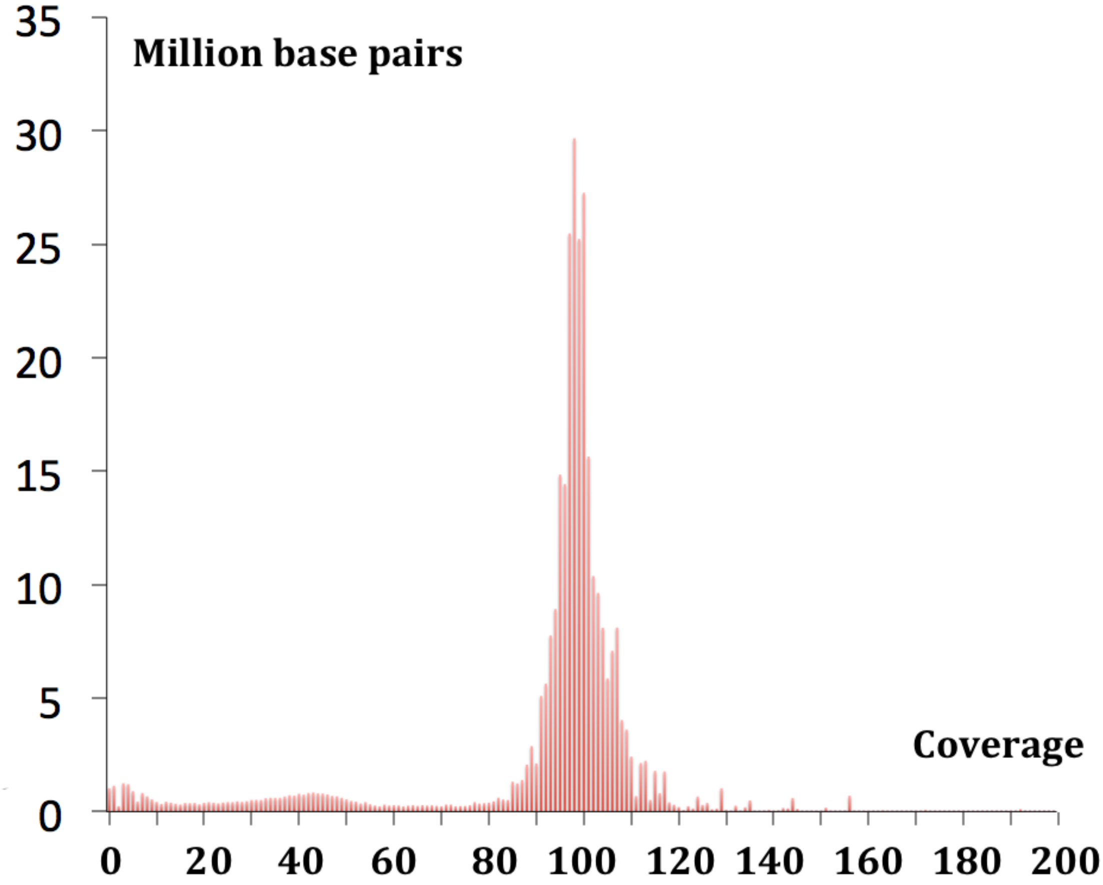

Figure S1. Average coverage for scaffolds from the *Lerema accius* assembly_V0 (the result directly obtained from the Platanus assembler). The peak at 44-fold coverage is likely dominated by short scaffolds, which correspond to highly heterozygous regions that were not merged to the equivalent segments in the homologous chromosomes.

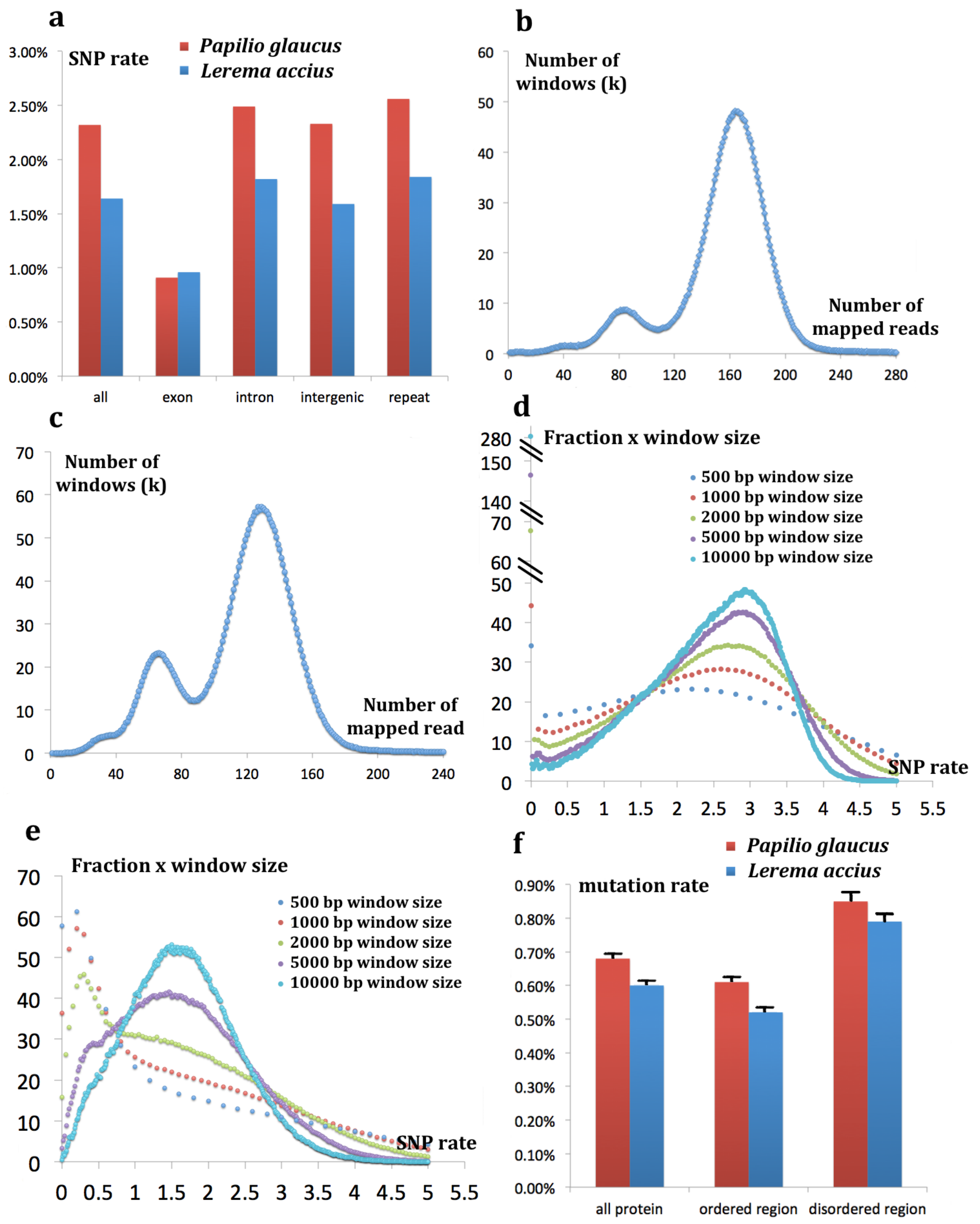

Figure S2 Comparison of the SNP distribution in the *Lerema accius* and *Papilio glaucus* genomes.

(a) The overall SNP rate in different regions of the *Lerema accius* (blue bars) and *Papilio glaucus* (red bars) genomes. (b) Histogram of the numbers of reads mapped to 100 bp non-overlapping windows in the *Papilio glaucus* genome. (c) Histogram of the numbers of reads mapped to 100 bp non-overlapping windows in the *Lerema accius* genome. In both (b) and (c), the peak on the left represents highly heterozygous regions in the genome where the equivalent regions in the homologous chromosomes cannot be merged by the assembler due to low levels of sequence similarity. (d) Histogram of SNP rates in overlapping windows of different sizes in the *Papilio glaucus* genome. (e) Histogram of SNP rates in overlapping windows of different sizes in the *Lerema accius* genome. (f) Overall substitution rate in the disordered and ordered protein-coding regions of proteins from *Lerema accius* (blue bars) and *Papilio glaucus* (red bars). In both species, the mutation rate in disordered regions is significantly higher (the confidence level > 99.9%) than that in ordered regions. The error bars are estimated from the standard deviation of substitution rate in individual proteins.

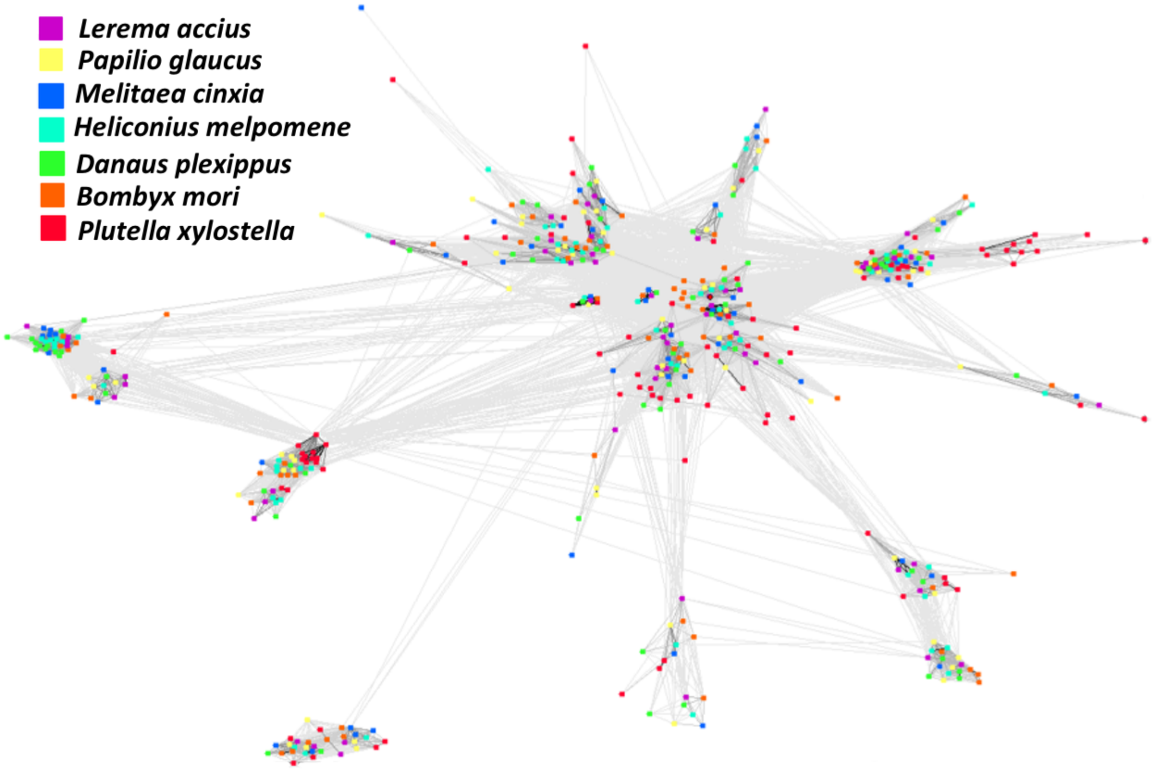

Figure S3 Clustering of odorant receptors from Lepidoptera genomes by sequence similarity. Each square represent one odorant receptor and its color shows the species it comes from. The grey lines connect pairs of highly similar odorant receptors with e-value smaller than 1e-30 in a pairwise BLAST comparison. This figure is generated with CLANS with a p-value (e-value) of 1e-30, i.e. only pairs with e-value lower than 1e-30 in pairwise BLAST comparisons will attract each other in the process of clustering.

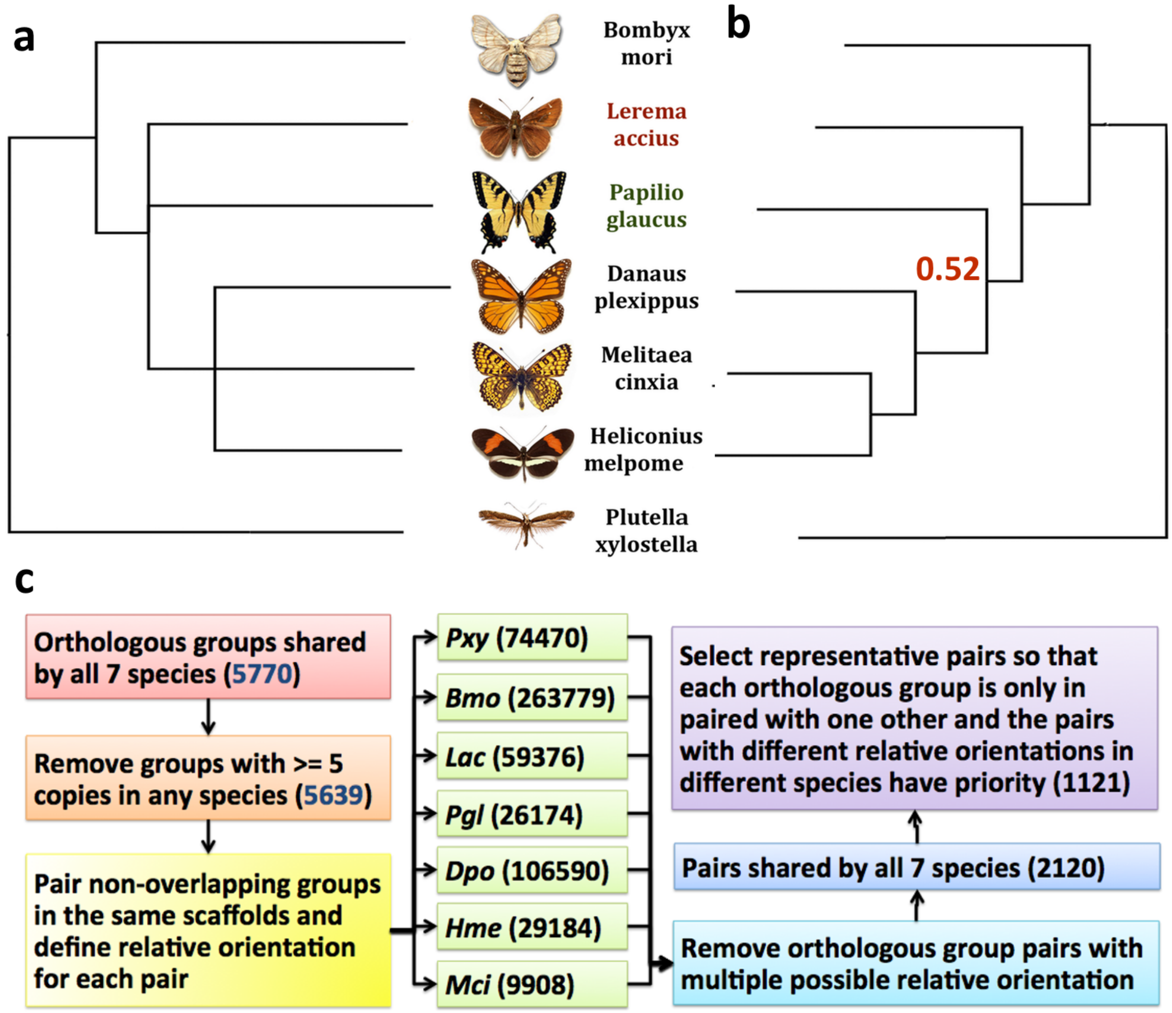

Figure S4. Phylogenetic analysis of Lepidoptera species. (a) 50%-majority-rule consensus tree of the maximal likelihood trees constructed by RAxML on alignments of individual proteins that have more than 100 confidently aligned positions. (b) Consensus of the better-supported tree inferred from PhyloBayes analysis on alignments of individual proteins with more that have more than 100 confidently aligned positions. In the PhyloBayes analysis, the tree topology was constrained to either of the two possible topologies under debate: (((((*Melitaea cinxia*, *Heliconius melpomene*), *Danaus plexippus*), *Lerema accius*), *Papilio glaucus*), *Bombyx mori*, *Plutella xylostella*) or (((((*Melitaea cinxia*, *Heliconius melpomene*), *Danaus plexippus*), *Papilio glaucus*), *Lerema accius*), *Bombyx mori*, *Plutella xylostella*). (c) Procedure of identifying gene pairs to perform phylogenetic analysis based on gene re-arrangement.

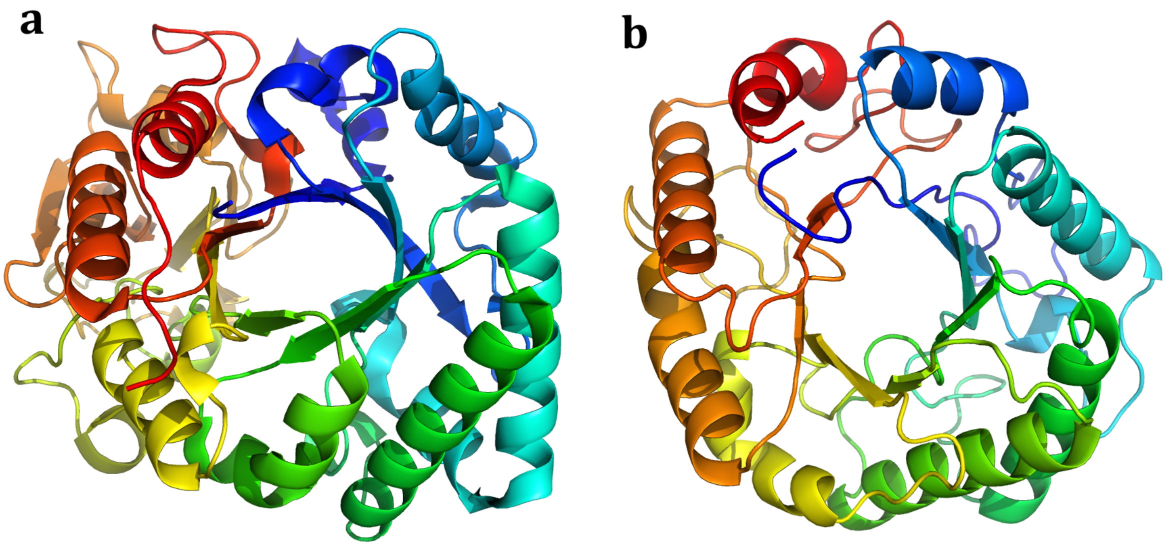

Figure S5. Three dimensional structures of endochitinase and cellulase. (a) A representative structure model of endochitinase in *Lerema accius* (template PDB id: 3WL1); (b) A representative structure of cellulase from fungi (PDB id: 1H1N).

Extended Experimental Procedures

S1 Sequencing library preparation protocol

S1.1 Genomic DNA extraction

The wings and abdomen of a freshly caught male *Lerema accius* (USA: Texas: Dallas County, Dallas, White Rock Lake, Olive Shapiro Park, 10-Nov-2013, GPS: 32.8621, -96.7305, elevation: 141 m) were removed and preserved. The rest were used to extract genomic DNA with ChargeSwitch gDNA mini tissue kit following the manufacturer's protocol with modifications.

A. Lysis

Divide sample into 2 pieces, and do the following steps for each piece:

Cut the tissue thoroughly into less than 1mm^3 pieces with a scalpel on a Petri dish;
Add 0.5 ml lysis buffer (L15) to the Petri dish and transfer the tissue to a 1.5 ml tube;
Wash the Petri dish with 0.5 ml lysis buffer and transfer the wash to the same tube;
Add 30 µl Proteinase K (20 mg/ml), flip the tube to mix and incubate at 55 °C overnight;
Add 20 µl RNase A (5 mg/ml), vortex and incubate at room temperature for 10 minutes.

B. Bind DNA

Add 120 µl of Purification Buffer (N5) to each tube and vortex to mix;
Resuspend the magnetic beads and add 100 µl to the each tube;
Flip the tube to mix and incubate at room temperature for 10 minutes;
Place the tube in the MagnaRack for 2 minutes and discard the supernatant.

C. Wash beads

Wash the beads twice and for each time:

Remove the tube from the MagnaRack and add 1 ml of Wash Buffer (W12);
Gently pipet up and down to resuspend the beads;
Place the tube in the MagnaRack for 2 minutes and discard the supernatant.

D. Elute DNA

Elute the DNA from the beads four times to increase the yield, and for each time:

Remove the tube from the MagnaRack and add 100 µl of Elution Buffer (E5);
Pipet gently to resuspend the beads and incubate at 37°C for 5 minutes;
Place the tube on the MagnaRack for 2 minutes;
Transfer the supernatant containing the purified DNA to a clean tube.

E. Quantify the amount of DNA

Use the Qubit dsDNA HS Assay Kit and Qubit fluorometer to measure the concentration of DNA following the manufacturer's protocol. We obtained approximately 20 µg of DNA. Check

the quality of genomic DNA using the E-gel 0.8% Agarose gel. One should expect to obtain long DNA fragments (about 40 kb) at this step, and this is necessary for the subsequent steps.

S1.2 Total RNA extraction

After removing the wings and abdomen, a freshly caught *Lerema accius* adult was preserved in *RNAlater* solution. Additionally, a pupa reared from a wild-collected caterpillar was preserved in *RNAlater* solution too. RNA was extracted from these two specimens using QIAGEN RNeasy plus mini kit following the manufacturer's protocol.

A. Homogenize the sample

Take about 10 mg tissue to a 1.5 ml tube and freeze the sample in liquid nitrogen;
Use a small pestle to grind the sample in the tube thoroughly and add 600 µl RLT buffer;
Load the lysis onto a QIAshredder homogenizer and centrifuge for 4 min at 15,000 rcf;

B. Bind DNA

Collect the flow-through in the collection tube from last step to the gDNA eliminating column (be careful to avoid the precipitant);
Centrifuge at 12,000 rcf for 30 s;

C. Bind RNA

Collect the flow-through from last step to a new 1.5 ml tube;
Add 0.6 ml (the same as the volume of RLT buffer used in step A.) 70% ethanol and mix;
Transfer no more than 0.7 ml solution to the RNA binding column (for each time) and spin at 12,000 rcf for 30 s, remove the flow-through and discard;
Repeat these steps until entire volume is processed through the RNA binding column.

D. Wash RNA

Add 700 µl buffer RW1 to the RNA column, spin at 12,000 rpm for 30s, and discard the flow-through;
Add 500 µl buffer RPE to the RNA column and spin at 12,000 rpm for 30s;
Add another 500 µl buffer RPE to the RNA column, spin at 12,000 rpm for 30 s, and discard the flow-through;
Transfer the column to a new collection tube, spin at 13,000 rpm for 2 min to dry it;

E. Elute RNA

Transfer the column to a RNA collection tube;
Add 30 µl nuclease-free water to the column, spin at 13,000 rpm for 30 s;
Add again 30 µl nuclease-free water to the column, spin at 13,000 rpm for 1 min.

F. Quantify the amount of RNA

Use the Qubit RNA HS Assay Kit and Qubit fluorometer and follow the manufacturer's protocol to measure the concentration of RNA. From 10 mg *RNAlater* treated tissue, we obtained approximately 4 µg of RNA. Check the quality of RNA using the E-gel 1% Agarose gel.

S1.3 Paired-end library preparation protocol

We prepared 250 bp and 500 bp paired-end libraries following a protocol similar to the Illumina TruSeq DNA sample preparation guide. For each paired-end library, approximately 500 ng genomic DNA was used.

A. Fragmentation

Material: Covaris S220 Focused-ultrasonicator, Covaris microTUBE and genomic DNA.
Parameters for the 250 bp library:

Intensity: 5
Duty cycle: 10%
Cycles per burst: 200
Treatment time: 90s
Volume: 50 µl
Temperature: 7°C

Parameters for 500 bp library:

Intensity: 5
Duty cycle: 5%
Cycles per burst: 200
Treatment time: 35s
Volume: 50 µl
Temperature: 7°C

B. End repair

Material: NEBNext End Repair Module and fragmented DNA.
Prepare the reaction in a 0.5 ml PCR tube:

50 µl fragmented DNA
35 µl sterile H_2O
10 µl End Repair Reaction Buffer (10X)
5µl End Repair Enzyme Mix.

Incubate at 20°C for 30 min and keep at 4°C for 30 min. Purify DNA with Ampure XP beads (1.8x volume) and elute 2 times in a total volume of 40 µl.

C. dA-tailing

Material: NEBNext dA-Tailing Module and end-repaired DNA.
Prepare the reaction in a 0.5 ml PCR tube:

40 µl end-repaired DNA
2 µl sterile H_2O

5 µl dA-Tailing Reaction Buffer (10X)
3 µl Klenow fragment

Incubate at 37°C for 30 min. Purify DNA with Ampure XP beads (1.8x volume) and elute 2 times in a total volume of 33 µl.

D. Adapters Ligation

Material: NEBNext Quick Ligation Module, Illumina TruSeq adapters and dA-tailed DNA. Adapters with different indices are needed for different libraries if they will be sequenced on the same lane.

Prepare the reaction in a 0.5 ml PCR tube:

33 µl dA-tailed DNA
2 µl TruSeq adapter
10 µl Quick Ligation Reaction Buffer (5X)
5 µl T4 Quick Ligase

Incubate at 20°C for 30 min. Purify DNA with Zymo DNA cleanup and concentrator-5 kit and elute twice in a total volume of 50 µl.

To remove the adapters and adapter dimers, purify the DNA again with Ampure XP beads (0.8X volume) and elute 2 times in a total volume of 35 µl

E. PCR amplification

Material: PCR Primer Cocktail, PCR Master Mix from TruSeq DNA sample Prep V2 kit, and adapter-ligated DNA.

Prepare the reaction in a 0.5 ml PCR tube:

5 µl PCR primer cocktail
15 µl PCR master mix
35 µl adapter-ligated DNA

Do PCR in a thermal cycler with a heated lid using the following program:

98°C for 30s
8 cycles of:
  98°C for 10s
  60°C for 30s
  72°C for 30s
72°C for 5 min
Hold at 4°C

Purify DNA with Ampure XP beads (1.8x volume) and elute twice in a total volume of 40 µl.

F. Size selection

Material: E-Gel EX 2% Agarose gel with the E-gel base and Trackit 50 bp DNA ladder from Invitrogen and PCR amplified DNA.

Distribute the PCR product into 4 lanes and dissect the band (about 4mm) at the desired fragment size. Recover the DNA from the gel using the Zymoclean gel DNA recovery kit following the manufacturer's protocol and elute to a final volume of 20 µl to obtain the final library.

S1.4 Mate pair library preparation protocol

We prepared 2 kb, 6 kb and 15 kb mate pair libraries using a modified version of a previously published mate pair library preparation protocol[1]. For the 2 kb, 6 kb and 15 kb libraries, about 1.7 µg, 3 µg and 7.2 µg genomic DNA was used, respectively.

A. Fragmentation 1

Material: Covaris S220 Focused-ultrasonicator, genomic DNA, Covaris miniTUBE (white) and Covaris gTUBE.

For a 2 kb library, prepare 1-2 µg DNA in 200 µl solution and shear DNA with Covaris S2 equipment in Covaris miniTUBE (white) using the following parameters:

Temperature: 7°C
Duty factor: 20%
Peak incident Power: 3
Cycles per burst: 1000
Treatment time: 15 min

For a 6kb library, prepare 2-4 µg DNA in 150 µl solution and shear DNA using Covaris gTUBE at eppendorf 5415R centrifuge under the following condition:

Speed: 12000 rpm
Temperature: 20 °C
Treatment time: 30s and then flip the tube, treat for another 30s

For a 15 kb library, prepare 6-8 µg DNA in 150 µl solution and shear DNA using Covaris gTUBE and eppendorf 5415R centrifuge under the following condition:

Speed: 5500 rpm
Temperature: 20 °C
Treatment time: 1 min and then flip the tube, treat for another 1 min

Purify DNA with Ampure XP beads. For 2 kb fragment, add 140 µl beads (0.7x volume); for 6 kb and 15 kb fragments, add 75 µl beads (0.5x volume). Elute with Zymo Zyppy elution buffer twice at 37 °C for 10 min in a total volume of 85 µl (add 44 µl each time).

B. End Repair 1

Material: NEBNext End Repair Module and fragmented DNA.

Prepare the reaction in a 0.5 ml PCR tube:

85 µl fragmented DNA
10 µl End Repair Reaction Buffer (10X)
5µl End Repair Enzyme Mix

Incubate at 20°C for 30 min and keep at 4°C for 30 min. Purify DNA with Ampure XP beads (0.7x volume for 2 kb library and 0.5x volume for 6 kb and 15 kb libraries). Elute with Zymo Zyppy elution buffer twice at 37 °C for 10 min in a total volume of 42 µl (add 22 µl each time).

C. **dA-tailing 1**

Material: NEBNext dA-Tailing Module and end-repaired DNA.
Prepare the reaction in a 0.5 ml PCR tube:

42 µl end-repaired DNA
5 µl dA-Tailing Reaction Buffer (10X)
3 µl Klenow fragment

Incubate at 20°C for 30 min and keep at 4°C for 30 min. Purify DNA with Ampure XP beads (0.7x volume for 2 kb library and 0.5x volume for 6 kb and 15 kb libraries). Elute with Zymo Zyppy elution buffer twice at 37 °C for 10 min in a total volume of 60 µl (add 31 µl each time).

D. **Ligation to circularization adapters**

Material: NEBNext Quick Ligation Module and the circularization adapters with loxP sites (customized DNA oligos from Integrated DNA Technology):

loxP1 double-stranded DNA oligo:
forward strand (with biotin label):
5’ CGATAACTTCGTATAATGTATGCTATACGAAGT(Bio-dT)ATTACGT 3’
reverse strand (with 5’ phosphate):
5’ (5Phos)CGTAATAACTTCGTATAGCATACATTATACGAAGTTATCGACC 3’
loxP2 double-stranded DNA oligo:
forward strand (with biotin label):
5’ GCATAACTTCGTATAGCATACATTATACGAAGT(Bio-dT)ATACGAT 3’
reverse strand (with 5’ phosphate):
5’ (5Phos)TCGTATAACTTCGTATAATGTATGCTATACGAAGTTATGCACC 3’

Prepare the reaction in a 0.5 ml PCR tube (mix annealed loxP1 with annealed loxP2 first):

60 µl dA-tailed DNA
2 µl annealed loxP1 adapter (50 µM)
2 µl annealed loxP2 adapter (50 µM)
20 µl Quick Ligation Reaction Buffer (5X)
10 µl T4 Quick Ligase

Incubate at 20°C for 30 min. Purify immediately after the incubation (prevent further ligation that may lead to hybrid) with Zymo DNA cleanup and concentrator-5 kit. To maximize the yield, bind each sample twice with 2 columns and elute each column twice in a total volume of 40 µl (add 21 µl each time).

E. **Fill-in reaction**

Material: NEB Bst DNA Polymerase, Large Fragment (8 U/µl, came with 10× ThermoPol Buffer) and dNTP Mix (10 mM each)
Prepare the reaction in a 0.5 ml PCR tube:

40 µl Circularization-adapted DNA (already in tube)
5 µl 10× ThermoPol Buffer
2 µl dNTP Mix (10 mM each)
3 µl Bst DNA Polymerase, Large Fragment (8 U/µl)

Incubate the fill-in reaction at 50°C for 15 minutes. Purify DNA with Ampure XP beads (0.7x volume for 2 kb library and 0.5x volume for 6 kb and 15 kb libraries). Elute with Zymo Zyppy elution buffer twice at 37 °C for 10 min in a total volume of 36 µl (add 19 µl each time).

F. Size selection

Material: E-gel EX 1% Agarose gel with the E-gel base, TrackIt 1kb DNA ladder and adapter-ligated DNA.
Distribute the PCR product in 4 lanes and run the gel until the DNA ladder is well separated. Dissect the band (about 1 cm to include most of the long fragments) at the desired length. Recover the DNA from the gel using the Zymoclean gel DNA recovery kit (ADB buffer volume to dissolve the gel: 1.5x for over 10 kb fragments and 2x for others) and elute twice to a final volume of 40 µl.

G. Concentration measurement

Material: Qubit dsDNA HS Assay Kit and Qubit fluorometer.
Follow the manufacturer's protocol to measure the concentration.
Note that in order to be successful with the following procedure, we recommend at least 400 ng DNA for the 2 kb library, 600 ng for the 6 kb library and 800 ng for the 15 kb library at this step. One can lose quite a lot of DNA during the processes above, so it is necessary to start with more DNA or to do the DNA purification steps with great care. Since we had a limited amount of DNA from only a piece of muscle of a single specimen, we performed the purification for each step very carefully (usually we bind 2-3 times and elute 3 times to increase the efficiency). We had about 40% yield for 2 kb and 6 kb libraries and 25% yield for the 15 kb library. In general, one can expect about 30% yield for libraries below 10 kb and less (15% - 20%) for longer ones.

H. Circularization and Digestion

Material: Cre recombinase (with buffer), Plasmid-Safe ATP dependent DNase (with 25 mM ATP) and *E coli*. Exonuclease I.
Dilute the adapter-ligated and size-selected DNA to a 2.5 ng/µl concentration. Assume the volume of DNA is 80x µL (x indicates an unknown number). Prepare the reaction in a 0.5 ml PCR tube (the final concentration of DNA in the reaction mix will be 2 ng/µl):

80x µl DNA
10x µl Cre recombinase buffer (10X)
10x µl Cre recombinase (1U/µl)

Incubate in a Thermocycler with the following program:

37°C for 50 minutes
70°C for 10 minutes
4°C forever

Once the temperature has reached 4°C, immediately add the following reagents:

1.1x µl DTT (100mM)
4.4x µl ATP (100mM)
5x µl Plasmid-Safe ATP-Dependent DNase (10U/µl)
3x µl Exonuclease I (20U/µl)

Incubate in a Thermocycler with the following program:

37°C for 30 minutes

80°C for 20 minutes

4°C forever

Purify the DNA by cold ethanol precipitation (-20 °C overnight) in the presence of 0.3M sodium acetate. After precipitation and wash (70% cold ethanol), dissolve DNA in a 50 µl Zymo Zyppy elution buffer. Since the majority of DNA will not be successfully circularized and will be digested by ATP dependent DNase and Exonuclease I, the DNA concentration at this point will be so low that only ethanol precipitation can lead to an acceptable recovery rate of DNA.

I. Fragmentation 2

Material: Covaris S220 Focused-ultrasonicator, Covaris microTUBE and genomic DNA.

Parameters for all libraries:

Peak intensity: 175

Duty cycle: 5%

Cycles per burst: 200

Treatment time: 45s

Volume: 50 µl

Temperature: 7°C

J. Immobilization with Streptavidin beads

Material: Dynabeads M-280 Streptavidin coated beads, 2X B&W buffer and fragmented DNA.

Immobilize DNA to the beads through the following steps:

Resuspend the beads and transfer 20 µl beads to a 0.5 ml PCR tube;

Remove the supernatant and wash the beads twice in 50 µl 2X B&W buffer;

Remove the supernatant, and add 50 µl 2X B&W buffer and 50 µl of fragmented DNA to the tube;

Incubate at 20°C for 1 hour on a rotator;

Remove the supernatant;

Wash the beads 4 times with 100 µl 2X B&W buffer and 2 times with 100 µl Zymo Zyppy elution buffer;

Remove the buffer and immediately proceed to the next step.

K. End repair 2

Material: NEBNext End Repair Module and immobilized DNA from last step.

Prepare the reaction in a 0.5 ml PCR tube:

All immobilized DNA from the last step

42.5 µl ddH_2O

5 µl End Repair Reaction Buffer (10X)

2.5 µl End Repair Enzyme

Incubate at 20°C for 30 min and keep at 4°C for 30 min. Remove the supernatant, wash 4 times with 100 µl 2X B&W buffer and 2 times with 100 µl Zymo Zyppy elution buffer. Remove the buffer and immediately proceed to the next step.

L. dA-tailing 2

Material: NEBNext dA-Tailing Module and end-repaired DNA.
Prepare the reaction in a 0.5 ml PCR tube:

- All immobilized DNA from the last step
- 24.6 µl ddH_2O
- 3 µl dA-Tailing Reaction Buffer (10X)
- 2.4 µl Klenow fragment

Incubate at 37°C for 30 min. Proceed immediately to the next step without washing.

M. Ligation to TruSeq adapters

Material: NEBNext Quick Ligation Module, Illumina TruSeq adapters and dA-tailed DNA. Remember to use different adapters for different libraries if they will be sequenced on the same lane.
Prepare the reaction in a 0.5 ml PCR tube:

- 30 µl dA-tailing reaction mix from the last step
- 1 µl TruSeq adapter
- 4 µl ddH_2O
- 10 µl Quick Ligation Reaction Buffer (5X)
- 5 µl T4 Quick Ligase

Incubate at 20°C for 30 min. Remove the supernatant, wash 6 times with 100 µl 2X B&W buffer and 4 times with 100 µl Zymo Zyppy elution buffer. Remove the buffer and immediately proceed to the next step.

N. PCR amplification

Material: PCR Primer Cocktail, PCR Mater Mix from TruSeq DNA sample Prep V2 kit, and adapter-ligated DNA.
Prepare reaction in a 0.5 ml PCR tube:

- All immobilized DNA from the last step
- 5 µl PCR primer cocktail
- 10 µl PCR master mix
- 35 µl ddH_2O

Carry out PCR in a thermal cycler with a heated-lid using the following program:

- 98°C for 30s
- 13 cycles of:
    - 98°C for 10s
    - 60°C for 30s
    - 72°C for 30s
- 72°C for 5 min
- Hold at 4°C

Transfer the supernatant (the PCR products are in the solution, not on the beads) into another tube to perform purification with Ampure XP beads (1.0x volume) and elute in 20 µl Zymo Zyppy buffer to get the final library.

S1.5 RNA-seq library preparation protocol

We prepared RNA-seq libraries for RNA extracted from the specimens in adult (specimen info) and pupal stages (specimen info) using NEBNext Ultra RNA Library Prep Kit for Illumina following the manufacturer's protocol with minor modifications.

A. mRNA isolation

Additional material: NEBNext Poly(A) mRNA Magnetic Isolation Module (NEB #E7490).
Protocol:

Dilute the total RNA to a final volume of 50 μl and keep on ice;

Take 15 μl of NEBNext Oligo d(T) beads and wash the beads twice with 100 μl of RNA Binding Buffer (2×);

Resuspend the beads in 50 μl of RNA Binding Buffer (2×) and add the 50 μl of total RNA sample;

Incubate at 65 °C for 5 min, cool down to 4 °C and immediately proceed to the next steps;

Incubate at room temperature for 5 min, place the tube on the magnetic rack for 2 min and discard the supernatant;

Wash the beads twice with 200 μl of Wash Buffer;

Elute mRNA with 50 μl of Tris Buffer;

Incubate at 80 °C for 2 min, cool down to 25 °C and immediately proceed to the next steps;

Add 50 μl of RNA Binding Buffer (2×) to the sample and incubate the tube at room temperature for 5 min, place the tube on the magnetic rack for 2 min and discard the supernatant;

Wash the beads once with 200 μl of Wash Buffer;

Wash the beads once with 200 μl of Tris Buffer, and ensure to remove all the supernatant;

B. Fragmentation

Prepare 12μl First Strand Synthesis Reaction system in a tube:

4.8 μl NEBNext First Strand Synthesis Reaction Buffer (5×)
1.2 μl NEBNext Random Primers
6 μl Nuclease-free water

Add this First Strand Synthesis Reaction system to the beads from last step;
Incubate the sample at 94 °C for 8 minutes, and place the tube on the magnetic rack;
Collect the purified mRNA by transferring 10 μl of the supernatant to a 0.5 ml PCR tube;

C. First strand cDNA synthesis

Add the following reagents to the 10 μl fragmented DNA in First Strand Reaction system:

0.5 μl Murine RNase Inhibitor
8.5 μl ddH_2O
1 μl ProtoScript II Reverse Transcriptase

Incubate the sample the sample in a thermal cycler as follows:

10 minutes at 25 °C
50 minutes at 42 °C

15 minutes at 70 °C

Hold at 4 °C

Proceed immediately to the second strand cDNA synthesis reaction.

D. Second strand cDNA synthesis

Add the following reagents to the First Strand Synthesis reaction (20 µl):

48 µl ddH_2O

8 µl Second Strand Synthesis Reaction Buffer (10X)

4 µl Second Strand Synthesis Enzyme Mix

Incubate in a thermal cycler for 1 hour at 16 °C;

Purify the double-stranded cDNA using 1.8× Ampure XP beads and elute in a total volume of 55.5 µl;

E. End preparation

Add the following reagents to the 55.5 µl cDNA:

6.5 µl End Preparation Reaction Buffer (10×)

3 µl End Preparation Enzyme Mix.

Incubate in a thermal cycler as follows:

20 °C for 30 min

65 °C for 30 min

hold at 4 °C

Proceed immediately to the adaptor ligation step.

F. Adapter ligation

Additional material: NEBNext Multiplex Oligos for Illumina

Add the following reagents to the 65 µl of the product resulting from end preparation reaction, and:

15 µl Blunt/TA Ligase Master Mix

3 µl 10-fold (1:9) Diluted NEBNext Adaptor

Incubate at 20 °C for 15 min;

Add 3 µl USER Enzyme and mix well;

Incubate at 37 °C for 15 min.

G. Size selection

Additional material: Ampure XP beads

Add 25 µl of resuspended AMPure XP Beads to the 83 µl ligation reaction, mix well and incubate for 10 min;

Quickly spin the tube and place the tube on an appropriate magnetic stand for about 2 min;

Transfer the supernatant containing the DNA to a new tube and discard the beads;

Add another 25 µl resuspended AMPure XP Beads to the supernatant, mix well, and incubate for 10 min at room temperature;

Quickly spin the tube and place it on an appropriate magnetic stand for 2 minutes;

Discard the supernatant that contains unwanted DNA;

Wash the beads twice with 150 µl of 80 % freshly prepared ethanol on the magnetic stand;

Dry the beads for 5 min with open lid;

Elute the DNA twice with 10 mM Tris-HCl buffer to a total volume of 23 µl and transfer the eluted DNA to a new PCR tube.

H. PCR amplification

Add the following reagents to the size-selected DNA from the last step:

1 µl Index Primer

1 µl Universal Primer

25 µl NEBNext High-Fidelity 2× PCR Master Mix

Do PCR in a thermal cycler with a heated lid using the following program:

98 °C for 30s

8 cycles of:

98 °C for 10 s

65 °C for 30 s

72 °C for 30 s

72 °C for 5 min

Hold at 4 °C

Purify DNA with Ampure XP beads (0.9× volume) and elute twice to a total volume of 20 µl.

S1.6 Preparation for sequencing on the Illumina HiSeq platform

Measure the concentration of all the libraries by QPCR with the KAPA Library Quantification Kit for Illumina sequencing platforms following the manufacturer's protocol. We mixed 250 bp, 500 bp, 2 kb, 6 kb, 15 kb DNA libraries, RNA-seq library from the pupa and RNA-seq library from the adult to get the final library for sequencing with the relative molar concentration of each library being 40:20:8:4:3:20:10. We sequenced the RNA-seq library from the adult specimen at a lower coverage because its RNA was partially degraded.

The final library was sent to the genomics core facility at University of Texas Southwestern Medical Center to sequence 150 bp at both ends (PE150) with a rapid run on HiSeq1500.

S2 Genome assembly strategy

In the rest of this document, a line starting with $ indicates a command line used in that step to execute a certain program or script, and a line starting with * explains the command line. In the command lines, parameter values that should be changed for the specific cases are placed in square brackets "[]".

S2.1 Data processing and error correction

We obtained QSEQ format sequencing results from the genomics core facility, and processed them with in-house scripts to: (1) remove reads that did not pass the purity filter; (2) classify the reads according to the TruSeq adapter indices and (3) output the reads into FASTQ-format files. In addition, the mate pair libraries were processed by the Delox script[1] (loxP sequence: TCGTATAACTTCGTATAATGTATGCTATACGAAGTTATTACGT) to remove the loxP sequences (if any) from the reads and separate the true mate pair reads from paired-end reads. All sequence reads were then processed sequentially by the following procedures.

(1) mirabait from the MIRA package (MIRALIB version V3.4.0)[2] to remove reads contaminated by the TruSeq adapters and oligos (sequences stored in the file junk.fa) used in the sequencing reactions.

$ mirabait -ik 20 junk.fa [inputfile] [outputfile]

(2) fastq_quality_trimmer from the FASTX-Toolkits (V0.0.13)[3] to remove low quality (quality score < 20) portion at both ends and to discard reads shorter than 10 bp after trimming.

$ fastq_quality_trimmer -t 20 -l 10 -i [inputfile] -o [outputfile]

(3) JELLYFISH (version 1.1.2)[4] to obtain K-mer frequencies from reads in all the libraries.

$ jellyfish count -m [length of k-mer] -t 8 -s 10000000000 -c 8 --timing=jf.err --both-strands --min-quality=20 --stats=jf.stats [inputfiles]

$ jellyfish dump -ct mer_counts_0 > infiles.cts

$ cut -f 2 infiles.cts | sort -nrk 1 | uniq -c > [output_for_making_histogram]

A histogram of 18-mer frequency shows that a cutoff of 7 times (frequency of a 18-mer) can separate the peak dominated by 18-mers with and without sequencing errors. This cutoff and the 18-mer counts from JELLYFISH were used subsequently to perform error correction with QUAKE[5]. We chose 18-mer frequency to determine this cutoff following the recommendation here: http://www.cbcb.umd.edu/software/quake/faq.html. They suggest to determine the length of k-mer based on k =log(200G)/log(4), where G is the size of the genome.

To run QUAKE (version 0.3), we prepared 3 files in the current directory: (1) cutoff.txt which has the cutoff for k-mer frequency; (2) "infiles" contains the paths for all the reads; (3) "infiles.cts" contains the JELLYFISH k-mer counts for reads in "infiles". The command for running QUAKE is:

$ quake.py -f infiles --int --no_cut --no_count -k [size of k-mer] -p [number_of_CPUs]

Afterwards, we used an in-house script find the corresponding pair for each read. Reads whose pairs were removed in previous steps were combined into a separate single-end library. This data processing resulted in 9 libraries stored in 17 files that were used to assemble the genome: two paired-end libraries with insert sizes 250 bp and 500 bp, three paired-end

libraries, three true mate pair libraries from the 2 kb, 6 kb and 15 kb libraries, and a single-end library containing all reads without pairs. JELLYFISH was applied a second time to the reads after error correction to generate a histogram for 17-mer frequencies to compare with a similar graph used in the *Papilio glaucus* genome project.

S2.2 Genome assembly

We tested three genome assemblers, including the well-established SOAPdenovo2 (version 2.04-r240)[6], ALLPATH-LG (version r43762)[7] and a new software designed for highly heterozygous genomes, Platanus (version 1.2.1)[8]. Both SOAPdenovo2 and ALLPATH-LG produced genome assembly with low scaffold N50. Platanus keeps track of the coverage for assembled regions in every stage of the assembling process and uses this information to detect and merge divergent equivalent regions in homologous chromosomes[8]. This strategy is suitable for many insect genomes with small size (indicates small number of gene duplication events) and high heterozygosity.

The performance of Platanus depends on the user-selected parameters, especially those defining the identity cutoff for merging divergent equivalent regions from homologous chromosomes. These parameters should depend on the heterozygosity level of a genome. For the *Papilio glaucus* genome with an overall heterozygosity rate of approximately 2% (see S5.1 for details), assembling with the following parameters produced an initial assembly with N50 comparable to published Lepidoptera genomes and assembly size (405Mbp) close to what we estimated based on K-mer (373Mbp). We call this initial result assembly_V0, which is the basis for the *Papiilo glaucus* genome draft. The commands used to produce assembly_V0 with Platanus are:

$ platanus assemble -f [fastq files for paired-end libraries] -t [number_of_CPUs] -o accius -m 128 -u 0.2 -a 5 -t 60

$ platanus scaffold -c accius_contig.fa -b accius_contigBubble.fa -IP1 [fastq files for 250bp paired-end library] -IP2 [fastq files for 500bp paired-end library] -IP3 [fastq files for paired-end libraries separated from mate pair libraries] -OP4 [fastq files for 2kb mate pair library] -OP5 [fastq files for 6kb mate pair library] -OP6 [fastq files for 15kb mate pair library] -u 0.2 -t [number_of _CPUs] -o glaucus

$ platanus gap_close –c accius_scaffold.fa -IP1 [fastq files for 250bp paired-end library] -IP2 [fastq files for 500bp paired-end library] -IP3 [fastq files for paired-end libraries separated from mate pair libraries] -OP4 [fastq files for 2kb mate pair library] -OP5 [fastq files for 6kb mate pair library] -OP6 [fastq files for 15kb mate pair library] -ed 0.1 -t [number_of_CPUs] -o accius

S2.3 Post-assembly improvement

The reads from all libraries used by the assembler were mapped to assembly_V0 with Bowtie2[9] and the results were further processed by SAMtools[10]. This mapping allowed us to calculate the average coverage for each scaffold and revealed that there are regions in the genome with about half of the expected coverage. As shown in supplemental Figure S1, the distribution of scaffold-level coverage has two peaks and the peak with higher coverage (center

of this peak is about 98.5 fold coverage) corresponds to the expected coverage of a diploid genome.

We assumed that if we did not have highly heterozygous regions that were not merged together, the histogram of coverage from 0 to 135 (centered around 98.5) should be similar to a normal distribution with and the left shoulder decaying in a similar way as the right shoulder. As there are repeats in the genome, the left shoulder is expected to decay even faster. Base pairs with coverage from 99 to 124 accounts for more than 95% of all base pairs that fall into the right shoulder (coverage from 99 to 199). Therefore, coverage above 124 is significantly different (confidence level: 95%) from the expected coverage (98.5). If there were no heterozygosity problems (i.e., both shoulders are symmetric), the cutoffs for significantly lower coverage and significantly higher coverage should be centered around 98.5 fold as well. Therefore, we estimated the cutoff for significantly lower coverage as 98.5 * 2 – 124 = 73, suggesting that coverage less than 73 fold is significantly different (confidence level 95%) from the expected value for a diploid genome.

The scaffolds with low coverage were likely dominated by highly heterozygous regions that were not merged with the equivalent segments in homologous chromosomes. Therefore, scaffolds with coverage less than 73 were merged into other scaffolds if they could be nearly fully (coverage >90%, uncovered region < 500 bp) aligned to another low-coverage region in a longer scaffold with high sequence identity (>95%). For scaffolds with even lower coverage and smaller size (size < 1000 bp and coverage < 39 or size < 10000 bp and coverage < 20), we used a looser cutoff for identity (> 90%) to merge them into the longer scaffolds. The assembly after this step, namely assembly_V1, is the current genome assembly and is used for gene annotation and other analysis. The scripts used for this step are available at: http://prodata.swmed.edu/LepDB/.

A similar problem occurred in the initial *Heliconius melpomene* genome assembly made by the CABOG assembler, because CABOG is not designed to work with heterozygous genomes[11]. The authors for *Heliconius* genome project adopted a strategy similar to ours to improve the initial assembly and to remove the redundant scaffolds resulting from divergent equivalent regions from homologous chromosomes[12]. In the *Papilio glaucus* genome project carried out by us, we used a similar strategy to improve the assembly as well.

While the widely used genome assembler ALLPATH-LG discards all the scaffolds smaller than 1000 bp, Platanus keeps all scaffolds regardless of their length, resulting in a large number of scaffolds in genome assembly_V1 (52833). This number would be reduced to 7614 if all the scaffolds both shorter than 1000 bp and lacking annotated proteins were removed. However, we prefer to keep our genome as complete as possible, and thus we included all short scaffolds in the *Lerema accius* genome draft.

S3 Transcriptome assembly strategy

S3.1 Data processing

The RNAseq libraries for the specimens in adult and pupal stages contain 4.3 Gbp and 11.1 Gbp data respectively, which is sufficient for transcriptome assembly. Similar to the procedure described in S2.1, reads with contamination from TruSeq adapters and the low quality portion of reads were removed using mirabait and fastq_quality_trimmer before they were supplied to the assemblers.

S3.2 *De novo* assembly, reference-guided assembly and mapping to the genome

We applied three methods to assemble the transcriptomes:

(1) *de novo* assembly by Trinity (version r20140413p1)[13,14]

$ Trinity --output .[output directory] --seqType fq --JM 100G --normalize_reads --left [RNAseq reads_1 in fastq format] --right [RNAseq reads_2 in fastq format] --CPU 24

(2) reference guided assembly by TopHat[15] (v2.0.10) and Cufflinks[16] (v2.2.1)

$ bowtie2-build [genome in fasta format] [indexed genome base name]

$ tophat --read-edit-dist 5 --fusion-read-mismatches 3 --segment-mismatches 3 --read-mismatches 4 --read-gap-length 4 --output-dir [output directory] --read-realign-edit-dist 0 --mate-inner-dist 100 --mate-std-dev 50 --solexa1.3-quals --num-threads 32 --coverage-search --b2-sensitive --library-type fr-unstranded [indexed genome base name] [RNAseq reads_1 in fastq format],[RNAseq_reads_2 in fastq format],[RNAseq single-end reads in fastq format]

$ cufflinks -p [number of CPUs] [TopHat alignments in bam format]

$ gffread -w transcripts.fa -o transcripts.gff -g [genome assembly in fasta format] transcripts.gtf

(3) reference guided assembly by Trinity based on TopHat's alignments.

$ Trinity –output [output directory] --normalize_reads --genome [genome assembly in fasta format] --genome_guided_max_intron 100000 --genome_guided_sort_buffer 18G --seqType fq --JM 18G --genome_guided_use_bam [TopHat alignment in bam format] --left [RNAseq reads_1 in fastq format] --right [RNAseq reads_2 in fastq format] --CPU 6 --genome_guided_CPU 6 --GMAP_CPU 6

The results from all three methods were then integrated by Program to Assemble Spliced Alignments (PASA, version r20130907)[17,18] with the following commands:

$ cat [Trinity *de novo* assembly in fasta format] [Trinity genome guided assembly in fasta format] > [Trinity assemblies]

$ seqclean [Trinity assemblies] -c 8

$ accession_extractor.pl < [Trinity assemblies] > tdn.accs

$ Launch_PASA_pipeline.pl -c alignAssembly.config -C -R -g [genome assembly in fasta format] -t [Trinity assemblies after seqclean] -T -u [Trinity assemblies before seqclean] --TDN tdn.accs --cufflinks_gtf [Cufflinks result in gtf format] --ALIGNERS blat,gmap --CPU [number of CPUs]

S4 Genome assembly quality assessment

We obtained the most recent versions of published Lepidoptera genomes, including *Bombyx mori*, *Danaus plexippus*, *Heliconius melpomene*, *Melitaea cinxia*, *Papilio glaucus* and *Plutella xylostella*[12,19-27], and compared their quality to the *Lerema accius* genome. In addition to the continuity reflected by N50, completeness is another very important indicator of genome quality. We evaluated the completeness of these genomes by analyzing the coverage of independently obtained transcripts, Core Eukaryotic Genes Mapping Approach (CEGMA)[28] genes and the Cytoplasmic Ribosomal Proteins. The evaluation was done using the criteria that were used in the Monarch butterfly genome paper[24].

S4.1 Genome assembly quality assessment by the coverage of transcripts

We adopted the criterion used in the Monarch butterfly genome paper, and considered a transcript to be covered if the e-value of its best BLASTN[29] hit in the genome of the same species is smaller than 10^{-50}. The *de novo* assembled transcriptomes from two *Lerema accius* specimens were used to evaluate the completeness of the *Lerema accius* genome. 96.6% (43,989 out of a total 45,550) of transcripts from the adult specimen and 98.9% (47,797 out of 48,338) of transcripts from the specimen in the pupal stage meet the criterion, respectively. The RNA quality for the first specimen was relatively poor, and thus the assembled transcripts from that specimen were shorter (on average 1071 bp, whereas the average transcript length for the other specimen is 1140). And this poor quality of RNA-seq library may explains why transcripts from the first specimen was not covered by the genome as much as those from the second specimen. The number of transcripts assembled by Trinity is large, as many of them are redundant with several transcripts mapping to the same loci.

Similar statistics for *Danaus plexippus*, *Bombyx mori*, *Melitaea cinxia, Paplio glaucus* and *Plutella xyostella* were taken from other genome papers. *Plutella xylostella* genome shows a particularly poor level of completeness by this measurement. This can be partly attributed to the high level of variation in the *Plutella xylostella* population and possible poor quality of the RNA sequences. However, meanwhile, the *Plutella xylostella* is likely the least complete among the Lepidoptera genome and this incompleteness is supported by another, independent test aiming to identify Hox genes, in which 2 out of the 14 conserved Hox genes are missing in the *Plutella xylostella* genome, but are present in its transcriptomes.

S4.2 Genome assembly quality assessment by the coverage of CEGMA genes

The 457 core eukaryotic genes (CEGMA genes) from *Drosophila melanogaster* were used to evaluate the completeness of Lepidoptera genomes and a gene was considered to be covered by the genome if its best TBLASTN hit in the genome had an e-value lower than 10^{-5}. By this criterion, three CEGMA genes (0.7%) are missing in the *Lerema accius* genome. However, the orthologs of these three genes are consistently missing in all independently sequenced Lepidoptera genomes. Therefore, possibilities other than genome incompleteness are more likely responsible for their absence: (1) their sequences in Lepidoptera genomes diverged a lot from *Drosophila*; (2) they are made of short exons in Lepidoptera genomes; and (3) they are not essential and lost in Lepidoptera.

To test whether the scaffolds in the genome assemblies are long enough to completely cover most of the protein coding genes, for each CEGMA gene we calculated the percentage of residues that were covered by the most confident scaffold in the TBLASTN alignment. Judging by this criterion, *Lerema accius* is comparable to other genomes with average coverage of 86.6%. The modest coverage at the residue level is expected, due to the presence of short exons and the distant relationship between *Drosophila* and Lepidoptera.

S4.3 Genome assembly quality assessment by Cytoplasmic Ribosomal Proteins

We searched Flybase[30,31] with the term “ribosomal proteins” and selected 93 Cytoplasmic Ribosomal Proteins manually from the result. We considered a Cytoplasmic Ribosomal Protein to be present in a Lepidoptera genome if either its best TBLASTN hit in the genome sequence or its best BLASTP hit in the protein set had an e-value below 10^{-5}. *Lerema accius* genome is among the most complete ones based on this measurement, and only the Ribosomal protein L41 from *Drosophila* is seemingly not present in the genome. However, Ribosomal protein L41 is consistently missing in all independently sequenced Lepidoptera genomes, and thus other reasons, rather than the incompleteness of these genomes might instead account for this apparent absence of this CPR.

S5 Detection of SNPs in the genome

In order to directly compare the SNP distribution for the two highly heterozygous genomes, *Lerema accius* and *Papilio glaucus*, we detected and analyzed the SNPs in them using exactly the same set of methods.

S5.1 SNP detection

The reads from all the libraries used to assemble the genome were mapped to the genome assembly and positions with SNPs were detected using the Genome Analysis Toolkit[32,33] (GATK) with the following commands.

$ java -jar CreateSequenceDictionary.jar REFERENCE=[genome assembly in fasta format] OUTPUT=[genome assembly as a dictionary]

$ java -jar SortSam.jar INPUT=[Bowtie2 alignments in SAM format] OUTPUT=[step1 BAM format output] SORT_ORDER=coordinate

$ java -jar MarkDuplicates.jar INPUT=[step1 BAM format output] OUTPUT=[step2 BAM format output] METRICS_FILE=metrics.txt

$ java -jar AddOrReplaceReadGroups.jar I=[step2 BAM format output] O=[step3 BAM format output] SORT_ORDER=coordinate RGID=group1 RGLB=lib1 RGPL=illumina RGPU=unit1 RGSM=[genome assembly base name] CREATE_INDEX=True

$ java -jar BuildBamIndex.jar INPUT=[step3 BAM format output]

$ java -jar GenomeAnalysisTK.jar -T RealignerTargetCreator -fixMisencodedQuals -R [genome assembly in fasta format] -I [step3 BAM format output] -o target_intervals.list

$ java -jar GenomeAnalysisTK.jar -T IndelRealigner -fixMisencodedQuals -R [genome assembly in fasta format] -I [step3 BAM format output] -targetIntervals target_intervals.list -o [step4 BAM format output]

$ java -jar GenomeAnalysisTK.jar -fixMisencodedQuals -l INFO -R [genome assembly in fasta format] -T UnifiedGenotyper -I [step4 BAM format output] -o [SNP calls in VCF format] --output_mode EMIT_ALL_SITES

S5.2 Distribution of SNPs in different genomic regions

To analyze the distribution of SNPs, we divided the genome into different regions, i.e. exons, introns, repeats and intergenic regions. The percentage of SNPs in window of various sizes (500 bp, 1,000 bp, 2,000 bp, 5,000 bp, and 10,000 bp) was used to reflect this distribution. This analysis initially revealed a large portion of windows without SNPs and we suspected that a lot of them were from highly divergent regions between the two homologous chromosomes and therefore only the reads originated from one of the homologous chromosomes could be mapped. In order to rule out this effect, we counted the number of reads mapped to every overlapping 100 bp window in the genome and plotted the distribution. This distribution shows two peaks: in addition to the mean peak centered around the expected coverage for a diploid genome, the peak on its left likely comes from the divergent regions mentioned above. We focused on the regions whose coverage by the reads falls within the diploid peak and analyzed the SNP distribution in exons, introns, repeats and intergenic regions again.

S5.3 Proteins enriched in substitutions

We mapped all SNPs to protein coding genes and detected non-synonymous SNPs that cause substitutions in proteins. We also predicted disordered regions in proteins with ESpritz server34 and found that disordered regions are significantly more enriched in substitutions (supplemental Figure S2f), which is likely due to the fact that disordered regions are more tolerant to them[35]. Substitutions could be enriched in proteins containing large portion of disordered regions regardless the function of that protein and excluding disordered regions in this analysis could benefit the identification of functional groups of proteins that are enriched in substitutions.

We identified proteins with significantly more substitutions in the non-disordered regions using binomial tests (p = average substitution rate in all proteins excluding the non-disordered regions, m = number of substitutions in a protein excluding disordered regions, N = length of the exons of a protein excluding disordered regions). To avoid false discoveries simply due to the large number of statistical tests performed, we carried out False Discovery Rate tests and calculated the Q-values (maximal FDR level)[36]. We consider proteins with Q-values smaller than 0.1 to be significantly enriched in substitutions.

The GO terms[37] and their parental GO terms associated with these substitution-enriched proteins were extracted, counted and compared with a background of GO terms (and their parental GO terms) associated with all proteins (excluding the completely disordered proteins) of a species. Significantly enriched GO terms were selected with binomial tests (p = probability for the GO term to be associated with any protein of this species that are not completely disordered, m = number of substitution-enriched proteins associated with this GO term, N = number of proteins with significantly enriched substitutions).

The significantly enriched GO terms (P-value < 0.01) associated with substitution-enriched proteins in both *Lerema accius* and *Papilio glaucus* were compared and the common ones were extracted and the joint P-value a GO term's enrichment in substitution-enriched proteins was calculated as the product of the P-values for its enrichment in both species. These common GO-terms and the joint P-values were submitted to the REVIGO web server[38] to cluster GO terms by similarity in meaning and to visualize them.

S6 Identification and classification of repeats

S6.1 Construction of a species-specific repeat library

To annotate the repeats and transposable elements in the *Lerema accius* genomes, we first used the RepeatModeler (version 3.0.9)[39] pipeline to detect species-specific repeat families. This pipeline employs two *de novo* repeat predictors, RECON[40] and RepeatScout[41] to identify similar sequences that repeatedly appear in different loci of the genome. We used the following commands and the final product of RepeatModeler is a list of representative sequences of repeat families in the genome.

$ BuildDatabase -name [database name] [genome assembly base name]

$ RepeatModeler -database [database name]

In addition, mapping reads to the genome reveals regions with significantly high coverage. This is true for genomes assembled with different methods. It is likely that such regions correspond to repeats in the genome that have not yet diverged. Due to the high sequence similarity (approaching 100%), genome assemblers merge them. We used in-house scripts to identify these repeats based on the number of reads mapped in 100 bp windows in the genome (introduced in S5.2). As shown in supplemental Figure S2b, the histogram of the number of reads mapped to homozygous regions has a peak at 164 reads. We considered any 100 bp windows with more than 652 (four times of the peak value) mapped reads to be from repeats and we joined neighboring 100 bp windows satisfying this criterion to obtain the complete repeat sequences. The repeat sequences detected by the two methods above were submitted to the CENSOR[42] web server (http://www.girinst.org/censor/) to assign them to the repeat and transposable element classification hierarchy. These repeats, and their classification status, were included to construct a species-specific repeat library.

S6.2 Detection and masking of repeats in the genome

The species-specific repeat library and the repeats classified in RepBase[43] (V18.12) were used to identify and mask repeats in the *Papilio glaucus* genome by RepeatMasker (version 3.0.9)[44] with the following commands:

$ RepeatMasker -lib [species specific repeat library] -pa 32 -div 30 [genome assembly in fasta format]

$ RepeatMasker -species all -pa 32 -div 30 [genome assembly with repeats masked from the last step]

We used a diversity cutoff of 30% (-div 0.3) for RepeatMasker, and detected 278,478 simple and interspersed repeats that comprise 15.5% of the genome (83.4 Mbp). This value is lower than many other Lepidoptera genomes. However, the number of repeats one can identify is sensitive to the procedures and parameters used in the data analysis. Therefore, it is difficult to conclude whether the difference in the repeat content of these genomes is significant unless the repeats are identified with the same procedure.

S7 Gene annotation

We annotated the protein coding genes with a pipeline very similar to what is implemented in the Broad Institute[45]. In short, transcript-based, homology-based and *de novo* approaches were used to generate 15 different sets of gene annotations. These annotations were combined with EvidenceModeller (version r2012-06-25)[18] to generate consensus-based final predictions.

S7.1 Transcript-based gene annotation

As described in S3.2, we assembled the transcriptomes of two *Lerema accius* specimens using different pipelines. For each specimen, we obtained two sets of transcript-based annotations from (1) a pipeline containing TopHat and Cufflinks and (2) a more sophisticated pipeline that uses PASA to integrate the results from Trinity *de novo* assembly, Trinity reference-guided assembly and the result from TopHat and Cufflinks. In total, these approaches produced 4 sets of gene annotations.

S7.2 Homology-based gene annotation

The protein sets from four published Lepidoptera genomes (*Bombyx mori*, *Danaus plexippus*, *Heliconius melpomene*, and *Plutella xylostella*) and the *Drosophila melanogaster*[46] in Flybase, were used as references to annotate *Lerema accius* proteins with the exonerate (version 2.2.0) software[47]. For each reference protein, we used the following command to produce a homology-based gene annotation.

$ exonerate --model protein2genome --refine region –q [reference protein sequence in fasta format] -t [genome assembly in fasta format] -Q protein -T dna --showtargetgff yes --showalignment yes --percent 30 > [output]

We enforced an identity cutoff of 30% to reduce the number of imperfect gene models based on remote homologs. This approach produced 5 sets of gene annotations that are based on different reference organisms.

In addition, proteins from the entire UniRef90[48] (Mar. 2014) database were used as references to annotate genes. For this large data set, we used genblastG (version 1.39)[49], a new, faster and splicing-site aware software that is similar to and is claimed to work no worse than exonerate. For each reference protein, we used the following command:

$ genblast -p genblastg -q [reference protein sequence in fasta format] -t [genome assembly in fasta format] -g T -v 2 -c 0.5 -e 0.00001 -s 0 -o [output base name] -gff -cdna -pro

The parameters "-g T -v 2 -c 0.5 -e 0.00001 -s 0" were specified for genblastG to limit the number of gene models with poor support. This approach generated 329,753 redundant gene models (several of them could map to the same loci) and all of them were used as one set of gene annotations.

S7.3 *De novo* gene annotation

One essential step for *de novo* gene annotation is to train the predictors with confident gene annotations. We manually curated and selected 1427 confident gene models by

integrating the evidence from transcripts and homologs with the help of in-house scripts. For homology-based predictions, only those models based on *Drosophila melanogaster*, *Danaus plexippus* and *Bombyx mori* proteins were used, because much effort has been made on the annotation proteins in these species. A confident gene model in *Lerema accius* needs to satisfy the following criteria: (1) both the homology-based methods and the transcript-based methods consistently predict the splicing sites inside this gene; (2) the predicted gene is completely covered by a transcript; (3) the predicted gene has a standard translation initiation site and stop codon.

We implemented four *de novo* gene predictors, AUGUSTUS (version 2.6.1)[50], SNAP (version 2006-07-28)[51], Genemark (version 2.3c)[52] and GlimmerHMM (version 3.0.1)[53]. Genemark is able to train itself on the input whole genome data with the following command:

$ gm_es.pl --max_nnn 1000 [input genome assembly in fasta format]

Other gene predictors were trained with our manually selected good gene models following the instructions from each program.

For AUGUSTUS, we used the following commands (scripts are from AUGUSTUS package):

$ perl gff2gbSmallDNA.pl [curated gene models in GFF format] [genome assembly in fasta format] 1000 [training set in Genbank format]

$ perl new_species.pl --species=[species name]

$ perl optimize_augustus.pl --cpus=32 --species=[species name] [curated gene models in Genbank format]

$ etraining --species=Papilio_glaucus [curated gene models in Genbank format]

For SNAP, we used the following commands (linux commands or scripts from SNAP package):

$ perl gff2zff.pl < [curated gene model in GFF format] > [training set in ZFF format]

* prepare the scaffold sequences in the same order as they show up in file [training set in ZFF format] and store them in file [sequences for training]

$ fathom [training set in ZFF format] [sequences for training] -gene-stats

$ fathom [training set in ZFF format] [sequences for training] -validate

$ fathom [training set in ZFF format] [sequences for training] -categorize 1000

$ fathom uni.ann uni.dna -export 1000 -plus

$ mkdir params

$ cd params/

$ forge ../export.ann ../export.dna

$ cd ..

$ hmm-assembler.pl [species name] params/ > [SNAP trained parameters]

For GlimmerHMM, we used the following commands:

$ python zff2glim.py [training set in ZFF format] > [training set for GlimmerHMM]

$ trainGlimmerHMM [sequences for training] [training set for GlimmerHMM] -b 2

For GlimmerHMM and Genemark, we used the genome sequence without repeat masking as input, because they do not handle masked repeats properly. Maker[54], a widely used gene annotation pipeline, also uses a genome sequence without masking as an input to

Genemark. AUGUSTUS and SNAP's performance can be significantly improved if evidence-based (transcripts and homologs) gene predictions are supplied to them. We used the Maker[54] pipeline to obtain evidence-guided predictions from AUGUSTUS and SNAP and *de novo* predictions from Genemark. In addition, we supplied all evidence-based and *de novo* predictions to Maker, so that it could make consensus-based predictions. Predictions made by Maker are similar to those predicted by *de novo* predictors and we retained them as an additional set of *ab initio* predictions. Thus, we constructed 5 sets of gene annotations made by *de novo* predictors.

Although Maker, AUGUSTUS and SNAP use homology and transcript-based evidence to assist gene prediction, we still consider their predictions to be *de novo*, because all these programs consider intrinsic features of the genomic sequence, such as quality of the open reading frames and the presence of transcription and translation initiation sites, to make their predictions. Consideration of these intrinsic features is the essence of *de novo* gene prediction.

S7.4 Consensus-based final gene annotation

All 15 sets of gene predictions discussed above and the annotation of repeats were integrated by EvidenceModeller to make the final gene predictions. As recommended by the author, we weighted transcript-based predictions more than homology-based ones, and weighted *de novo* predictions the least. For predictions that tend to be more reliable, a higher weight was given[54,55]. The weights we assigned for all the annotation resources are:

```
PROTEIN	exonerate:Hm	3
PROTEIN	exonerate:Dp	10
PROTEIN	exonerate:Px	2
PROTEIN	exonerate:Dm	5
PROTEIN	exonerate:Bm	5
PROTEIN	genBlastG	5
TRANSCRIPT	Cufflinks:adult	4
TRANSCRIPT	Cufflinks:pupa	6
TRANSCRIPT	PASA:adult	8
TRANSCRIPT	PASA:pupa	12
ABINITIO_PREDICTION	maker	10
ABINITIO_PREDICTION	augustus	4
ABINITIO_PREDICTION	snap	3
ABINITIO_PREDICTION	genemark	2
ABINITIO_PREDICTION	GlimmerHMM	1
```

We used the following commands to get EvidenceModeller predictions:

*all repeats annotation in file "repeats.gff3", all the transcript-based annotations in file "transcript.gff3", all the homology-based annotations in file "homolog.gff3" and all the *de novo* prediction in "denovo.gff3"

$ perl partition_EVM_inputs.pl --genome [genome assembly in fasta format] --gene_predictions denovo.gff3 --protein_alignments homolog.gff3 --transcript_alignments transcript.gff3 --

repeats repeats.gff3 --segmentSize 2000000 --overlapSize 10000 --partition_listing partitions_list.out
$ perl write_EVM_commands.pl --genome [genome assembly in fasta format] --gene_predictions denovo.gff3 --protein_alignments homolog.gff3 --transcript_alignments transcript.gff3 --repeats repeats.gff3 --output_file_name evm.out --partitions partitions_list.out --weights [file with weights listed above] --search_long_introns 1 --re_search_intergenic 1 > commands.list
* carry out all the commands in "commands.list" on multiple CPUs.

For proteins and the protein families that were further analyzed in the manuscript, we manually curated the gene models from EvidenceModeller and modified a small number of gene models. This manual curation resulted in the detection of a few genes missed by EvidenceModeller, which we added to the final gene set.

S7.5 Prediction of protein function and additional features

The well-curated protein annotations in the Swissprot[56] database have been shown to be of high quality[57]. Therefore, we predicted the function of *Lerema accius* proteins by transferring annotations from the closest BLAST hit in Swissprot, requiring the e-value to be less than 10^{-5}. This approach annotated 11,197 proteins. In addition, for each protein, we identified its closest *Drosophila melanogaster* homolog in Flybase and detected confident homologs (e-value < 10^{-5}) for 11,792 proteins. This mapping provides a better description of the putative function for each protein by linking the rich information and literature associated with the *Drosophila* protein to the *Lerema accius* protein.

Finally, we applied the comprehensive pipeline, InterproScan (version 5.6)[58], to every *Papilio glaucus* protein to identify conserved protein domains[59-65] and functional motifs[63,66], to predict sequence features including coiled coil[67], transmembrane helices[68,69] and signal peptides[69,70], to detect homologous structures[71,72] that can be used for structure prediction, to assign *Papilio glaucus* proteins to protein families[59-65] and to map them to metabolic pathways[73-76]. For each protein, we ran InterproScan with the following command:
$ interproscan.sh -i [protein sequence in fasta format] -b result/ -dp -goterms –pa

The GO terms associated with the closest *Drosophila* homologs and the closest BLAST hit in Swissprot were transferred to the *Lerema accius* proteins. Together with GO terms annotated by InterproScan, we obtained associated GO term annotations for 12,112 proteins. Combining function description transferred from Swissprot entries and the GO term annotations, we were able to predict the functions of 12,283 *Lerema accius* proteins.

S8 Comparison of Lepidoptera genomes

S8.1 Identification of orthologs

We compared the *Lerema accius* protein set with the official protein sets from several published Lepidoptera genomes, including *Bombyx mori*, *Danaus plexippus*, *Heliconius melpomene, Melitaea cinxia, Papilio glaucus* and *Plutella xylostella*. We used OrthoMCL (version 2.0.9)[77] to identify the orthologous protein groups from these species. We followed the User Guide came with the OrthoMCL package. Briefly, after modifying the configure file "orthomcl.config" to indicate database names and login information for MySQL, the following commands were used:

$ orthomclInstallSchema my orthomcl.config install_schema.log

* for each species, combine the proteins sequences in one fasta format file. For each fasta file, do the following command:

$ orthomclAdjustFasta [species name abbreviation] [protein sequence in fasta format] [id_field]

* this command will produce input fasta files for the next step in "./compliantFasta" directory.

$ orthomclFilterFasta ./compliantFasta/ 10 20

* this step will produce filtered sequences from all species in goodProteins.fasta
* for all protein sequences in goodProteins.fasta, do All-against-All BLASTP comparison and save the results in goodProteins.blast

$ orthomclBlastParser goodProteins.blast ./compliantFasta >> similarSequences.txt

$ orthomclLoadBlast orthomcl.config similarSequences.txt

$ orthomclPairs orthomcl.config

$ orthomclDumpPairsFile orthomcl.config

$ mcl mclInput --abc -I 1.5 -o mclOutput

$ orthomclMclToGroups [prefix of group names] [starting number for group names] < mclOutput > groups.txt

S8.2 Analysis of Hox genes

Starting with all homeodomains from *Drosophila* in the HomeoDB[78], we identified all homeodomains in Lepidoptera genomes using BLASTP (e-value cutoff 0.001). We made a multiple sequence alignment of all the Lepidoptera homeodomains with Muscle[79]. Muscle attempts to cluster similar sequences together, and on the basis of this clustering, we manually clustered these homeodomains into orthologous groups. The clustering was trivial because homeodomain sequences in the same orthologous groups are frequently almost identical, except for 15 divergent ones that are mapped to the loci corresponding to the *Drosophila* Hox genes, *Zen* and *Zen2*. We then focused on the homeodomains from the Hox genes. We built an evolutionary tree for homeodomains in Hox genes using RAxML[80] with automatically selected model (-m PROTGAMMA) based on the sequence alignment made by MAFFT[81]. We mapped the homeodomains from Hox genes to the genomes and revealed that their order in different genomes is mostly conserved. The expansion of *Zen*-like genes is a common feature of all the Lepidoptera species[82] that we analyzed and there is an additional, significant expansion of *Zen*-like genes in *Bombyx mori*. However, their poor conservation compared to other Hox genes suggests that they may not play an important role and might even be pseudogenes.

S8.3 Identification of Odorant Receptors (OR)

Starting from the annotated odorant receptors from the *Bombyx mori*, *Heliconius melpomene* and *Danaus plexippus* genomes, we identified all the ORs in the annotated protein sets from these Lepidoptera genomes using reciprocal BLAST. Proteins encoded by the genome but missed in the protein sets were predicted with the help of genblastG and their relationship to the annotated ORs was validated with reciprocal BLAST. All the candidates identified by the automatic programs were further curated to remove short fragments (<200 aa) and false positive hits that do not detect odorant receptors as the top hit in a BLAST search against Flybase entries. Sequences of these odorant receptors were compared and clustered using CLANS[83] with the following command in a linux machine:

$ java -jar clans.jar -infile [fasta file with sequences] -cpu [number of cpus] -blastpath "[path to blast]/blastall -p blastp" -formatdbpath "[path to formatdb]/formatdb" -eval 1 -pval 0.1 > runlog

After obtaining the results for pairwise BLAST in the file "tmpblasthsp.txt", both this BLAST result file and the input fasta file were moved to a windows-based computer and the following command was carried out to cluster the sequences and visualize the results:

$ java -jar clans.jar -infile [fasta file with sequences]

S8.4 Identification of expanded gene families

Similar to what we did to Lac proteins, we annotated proteins in other Lepidoptera genomes by identifying their confident and closest homolog in Flybase and Swissprot and transferring their GO terms to the Lepidoptera proteins. We classified the Lepidoptera proteins in whole genomes into families on the basis of orthologous groups identified by OrthoMCL and the mapping of these proteins to the *Drosophila melanogaster* proteins in FlyBase by BLAST (e-value < 10^{-5}). If two OrthoMCL-defined orthologous groups overlapped in the *Drosophila* proteins to which they map, we merged them into a single protein family. This approach allowed us to group most proteins with the same function or highly similar functions together.

Two criteria were used to identity expanded gene families in *Lerema accius*: (1) *Lerema accius* must have more than one proteins from this family; (2) *Lerema accius* must have more proteins from this family than any other Lepidoptera species; (3) the total length of *Lerema accius* proteins in this family must be at least 1.5 times more than the total protein length for any other Lepidoptera; (4) the total length of *Lerema accius* proteins in this family must be at least 1.5 times longer than the average length of their closest homologs in Flybase. Proteins satisfying all these criteria are listed in Table S15 and ranked by the minimum of two gene expansion indices: (1) the ratio of *Lerema accius* protein number to the average protein number in other species (considering the average to be 1 if its actual value is smaller than 1) and (2) the ratio of *Lerema accius* protein total length to the average total length for other species (considering this average total length to be the average length of their closest homologs in Flybase if the former value is smaller than the latter).

S8.5 In-depth study of important gene expansion events

The most interesting and most confident gene expansion events were further investigated. For each family, the following steps were taken to ensure the inclusion of all relevant proteins: (1) search for homologs (e-value < 10^{-5}) in all Lepidoptera protein sets starting with all current members in a family; (2) annotate all hits by transferring the annotation from the best BLAST hit (e-value < 10^{-10}) in the Swissprot database; (3) remove the proteins that are remotely related and of different function based on BLAST statistics and function annotation; (4) use proteins remaining after step 3 to search against the genome sequences by genblastG to obtain additional proteins that were missed in the official gene sets; (5) group proteins from the previous two steps according to their genomic loci and at each loci, select the best (by length and similarity to other proteins) gene model and remove other redundant models. Usually, we preferred to select gene models that are the same as those in the official protein sets since such models might be supported by RNA sequences. However, we sometimes modified these gene models, e.g. by extending the coding sequence or by separating a fused protein into two, so that they became more consistent with orthologs in other species. All these steps were performed with in-house scripts combined with manual curation.

One problem with interpretation of highly heterozygous genome is that highly divergent alleles from homologous chromosome may still appear in the genome assembly as two segments and they can be misinterpreted as duplication. We confirmed that was not the case for the expanded gene families we studied here using the following two criteria: (1) the sequence identity between a pair of proteins should be below 95%; (2) the coverage of the coding genes by the sequence reads should be about the expected value for a diploid genome. Protein sequences from each protein family were aligned with MAFFT. Evolutionary trees were then built using RAxML with evolutionary models that are automatically determined by the program (-m PROTGAMMA) and visualized in FigTree.

S9 Phylogenetic analysis

S9.1 Phylogenetic analysis based on universal single-copy orthologs

2940 orthologous groups made of single-copy orthologs from all genomes were extracted from OrthoMCL output and used to build a phylogenetic tree. We built alignment for each orthologous group using both global sequence aligner MAFFT and local sequence aligner BLASTP. Positions that were consistently aligned by both aligners were extracted to obtain the confident alignment for each protein. All the alignments were concatenated and the aligned positions were randomly divided to 100 groups (each group contained more than 5,000 aligned positions). We repeated this procedure 10 times to obtain a total of 1,000 representative alignments for phylogenetic analysis. Additionally, 1991 out of the 2940 single-copy orthologous groups contain more than 100 consistently aligned positions. They were used as another data set for the same analysis.

We first used the maximal-likelihood method RAxML with the best model automatically selected by the program to construct phylogenetic trees for both random samples of the concatenated alignment and the alignments of individual gene. We provided a constraint tree (((*Melitaea cinxia*, *Heliconius melpomene*, *Danaus plexippus*), *Lerema accius*, *Papilio glaucus*), *Bombyx mori*, *Plutella xylostella*) to the program. In the tree topologies listed above and below, the first letter of the genus name and the first two letters of the species name were used to represent each species. This species grouped by the constraint tree should always form a clade based on previous phylogenetic analysis that used either morphological features or molecular data. We used this constraint to ensure a more efficient search aiming at resolving the uncertain relationships. An example command of running RAxML is like:

$ raxmlHPC-SSE3 -g [constraint tree] -m PROTGAMMAAUTO -s [input alignment] -n [basename of the result files] -p [random seed] &

We then used another, more elaborate Bayesian method PhyloBayes[84] with CAT model[85] to compare the two possible topologies under debate: (((((*Melitaea cinxia*, *Heliconius melpomene*), *Danaus plexippus*), *Lerema accius*), *Papilio glaucus*), *Bombyx mori*, *Plutella xylostella*) or (((((*Melitaea cinxia*, *Heliconius melpomene*), *Danaus plexippus*), *Papilio glaucus*), *Lerema accius*), *Bombyx mori*, *Plutella xylostella*). CAT is the infinite mixture model, in which it is assumed that aligned positions may belong to different **cat**egories, each undergoing substitution process in a distinctive manner. Comparing the posterior probabilities given the two topologies allowed us to select the tree topology that is better supported by the data for each alignment. An example command of running PhyloBayes is like:

$ pb -d [input alignment] -T [testing tree topology] -nchain 2 100 0.1 100 [basename for the result files]

S9.2 Phylogenetic analysis based on gene re-arrangement events

In addition, we used the frequencies of gene rearrangements to construct phylogenetic trees. As illustrated in supplemental Figure S4c, we started from 5770 orthologous families present in all 7 species and removed families with extensive gene duplications (more than 4

copies of a gene in any species), which resulted in 5639 families. In each species, we determined the relative genomic orientation for every pair of gene families on the same scaffold. There are four possible relative orientations: [a+, b+]; [a-, b-]; [a+, b-]; [a-, b+], where a and b are genes from two families and “+” and “-” indicate the DNA strand they are encoded on. Due to the limited continuity of draft genomes, relative orientations in all 7 species could be determined for 2120 such gene pairs. Then, we restricted the analysis to 1121 such pairs so that each family participated in only one pair. We used four letters (A, B, C, and D) to denote the relative orientations of family pairs, and expressed the arrangement of the 1121 pairs in each species by a string of these letters. These strings were used as input for PhyloBayes for tree construction. The numbers of differences between these strings were used as evolutionary distances between species to construct phylogenetic tree with BioNJ[86] method from the phylogeny.fr web server[87].

Supplemental References

1 Van Nieuwerburgh, F. *et al.* Illumina mate-paired DNA sequencing-library preparation using Cre-Lox recombination. *Nucleic acids research* **40**, e24 (2012).

2 Chevreux, B., Wetter, T. & Suhai, S. Genome Sequence Assembly Using Trace Signals and Additional Sequence Information. *Computer Science and Biology: Proceedings of the German Conference on Bioinformatics* **99**, 45-56 (1999).

3 http://hannonlab.cshl.edu/fastx_toolkit/.

4 Marcais, G. & Kingsford, C. A fast, lock-free approach for efficient parallel counting of occurrences of k-mers. *Bioinformatics* **27**, 764-770 (2011).

5 Kelley, D. R., Schatz, M. C. & Salzberg, S. L. Quake: quality-aware detection and correction of sequencing errors. *Genome biology* **11**, R116 (2010).

6 Luo, R. *et al.* SOAPdenovo2: an empirically improved memory-efficient short-read de novo assembler. *GigaScience* **1**, 18, doi:10.1186/2047-217X-1-18 (2012).

7 Gnerre, S. *et al.* High-quality draft assemblies of mammalian genomes from massively parallel sequence data. *Proceedings of the National Academy of Sciences of the United States of America* **108**, 1513-1518, doi:10.1073/pnas.1017351108 (2011).

8 Kajitani, R. *et al.* Efficient de novo assembly of highly heterozygous genomes from whole-genome shotgun short reads. *Genome research* **24**, 1384-1395 (2014).

9 Langmead, B. & Salzberg, S. L. Fast gapped-read alignment with Bowtie 2. *Nature methods* **9**, 357-359 (2012).

10 Li, H. *et al.* The Sequence Alignment/Map format and SAMtools. *Bioinformatics* **25**, 2078-2079 (2009).

11 Miller, J. R. *et al.* Aggressive assembly of pyrosequencing reads with mates. *Bioinformatics* **24**, 2818-2824, doi:10.1093/bioinformatics/btn548 (2008).

12 Heliconius Genome, C. Butterfly genome reveals promiscuous exchange of mimicry adaptations among species. *Nature* **487**, 94-98 (2012).

13 Grabherr, M. G. *et al.* Full-length transcriptome assembly from RNA-Seq data without a reference genome. *Nature biotechnology* **29**, 644-652, doi:10.1038/nbt.1883 (2011).

14 Haas, B. J. *et al.* De novo transcript sequence reconstruction from RNA-seq using the Trinity platform for reference generation and analysis. *Nature protocols* **8**, 1494-1512 (2013).

15 Kim, D. *et al.* TopHat2: accurate alignment of transcriptomes in the presence of insertions, deletions and gene fusions. *Genome biology* **14**, R36 (2013).

16 Roberts, A., Pimentel, H., Trapnell, C. & Pachter, L. Identification of novel transcripts in annotated genomes using RNA-Seq. *Bioinformatics* **27**, 2325-2329 (2011).

17 Haas, B. J. *et al.* Improving the Arabidopsis genome annotation using maximal transcript alignment assemblies. *Nucleic acids research* **31**, 5654-5666 (2003).

18 Haas, B. J. *et al.* Automated eukaryotic gene structure annotation using EVidenceModeler and the Program to Assemble Spliced Alignments. *Genome biology* **9**, R7 (2008).

19 Duan, J. *et al.* SilkDB v2.0: a platform for silkworm (Bombyx mori) genome biology. *Nucleic acids research* **38**, D453-456 (2010).
20 Xia, Q. *et al.* A draft sequence for the genome of the domesticated silkworm (Bombyx mori). *Science* **306**, 1937-1940 (2004).
21 International Silkworm Genome, C. The genome of a lepidopteran model insect, the silkworm Bombyx mori. *Insect biochemistry and molecular biology* **38**, 1036-1045 (2008).
22 You, M. *et al.* A heterozygous moth genome provides insights into herbivory and detoxification. *Nature genetics* **45**, 220-225 (2013).
23 Tang, W. *et al.* DBM-DB: the diamondback moth genome database. *Database : the journal of biological databases and curation* **2014**, bat087 (2014).
24 Zhan, S., Merlin, C., Boore, J. L. & Reppert, S. M. The monarch butterfly genome yields insights into long-distance migration. *Cell* **147**, 1171-1185 (2011).
25 Zhan, S. & Reppert, S. M. MonarchBase: the monarch butterfly genome database. *Nucleic acids research* **41**, D758-763 (2013).
26 Ahola, V. *et al.* The Glanville fritillary genome retains an ancient karyotype and reveals selective chromosomal fusions in Lepidoptera. *Nature communications* **5**, 4737, doi:10.1038/ncomms5737 (2014).
27 Cong, Q., Borek, D., Otwinowski, Z. & Grishin, N. V. Tiger Swallowtail Genome Reveals Mechanisms for Speciation and Caterpillar Chemical Defense. *Cell reports*, doi:10.1016/j.celrep.2015.01.026 (2015).
28 Parra, G., Bradnam, K. & Korf, I. CEGMA: a pipeline to accurately annotate core genes in eukaryotic genomes. *Bioinformatics* **23**, 1061-1067 (2007).
29 Altschul, S. F., Gish, W., Miller, W., Myers, E. W. & Lipman, D. J. Basic local alignment search tool. *Journal of molecular biology* **215**, 403-410, doi:10.1016/S0022-2836(05)80360-2 (1990).
30 Ashburner, M. & Drysdale, R. FlyBase--the Drosophila genetic database. *Development* **120**, 2077-2079 (1994).
31 St Pierre, S. E., Ponting, L., Stefancsik, R., McQuilton, P. & FlyBase, C. FlyBase 102--advanced approaches to interrogating FlyBase. *Nucleic acids research* **42**, D780-788 (2014).
32 McKenna, A. *et al.* The Genome Analysis Toolkit: a MapReduce framework for analyzing next-generation DNA sequencing data. *Genome research* **20**, 1297-1303, doi:10.1101/gr.107524.110 (2010).
33 DePristo, M. A. *et al.* A framework for variation discovery and genotyping using next-generation DNA sequencing data. *Nature genetics* **43**, 491-498 (2011).
34 Walsh, I., Martin, A. J., Di Domenico, T. & Tosatto, S. C. ESpritz: accurate and fast prediction of protein disorder. *Bioinformatics* **28**, 503-509, doi:10.1093/bioinformatics/btr682 (2012).
35 Macossay-Castillo, M., Kosol, S., Tompa, P. & Pancsa, R. Synonymous constraint elements show a tendency to encode intrinsically disordered protein segments. *PLoS computational biology* **10**, e1003607, doi:10.1371/journal.pcbi.1003607 (2014).

36 Storey, J. D. & Tibshirani, R. Statistical significance for genomewide studies. *Proceedings of the National Academy of Sciences of the United States of America* **100**, 9440-9445 (2003).
37 Ashburner, M. *et al.* Gene ontology: tool for the unification of biology. The Gene Ontology Consortium. *Nature genetics* **25**, 25-29, doi:10.1038/75556 (2000).
38 Supek, F., Bosnjak, M., Skunca, N. & Smuc, T. REVIGO summarizes and visualizes long lists of gene ontology terms. *PloS one* **6**, e21800 (2011).
39 Smit, A. F. A. & Hubley, R. (http://www.repeatmasker.org) RepeatModeler Open-1.0. (2008-2010).
40 Bao, Z. & Eddy, S. R. Automated de novo identification of repeat sequence families in sequenced genomes. *Genome research* **12**, 1269-1276, doi:10.1101/gr.88502 (2002).
41 Price, A. L., Jones, N. C. & Pevzner, P. A. De novo identification of repeat families in large genomes. *Bioinformatics* **21 Suppl 1**, i351-358, doi:10.1093/bioinformatics/bti1018 (2005).
42 Jurka, J., Klonowski, P., Dagman, V. & Pelton, P. CENSOR--a program for identification and elimination of repetitive elements from DNA sequences. *Computers & chemistry* **20**, 119-121 (1996).
43 Jurka, J. *et al.* Repbase Update, a database of eukaryotic repetitive elements. *Cytogenetic and genome research* **110**, 462-467 (2005).
44 Smit, A. F. A., Hubley, R. & Green, P. (http://www.repeatmasker.org) RepeatMasker Open-3.0. (1996-2010).
45 Institute, B. (http://www.broadinstitute.org/annotation/genome/Geomyces_destructans/GeneFinding.html) Gene Finding Methods.
46 Misra, S. *et al.* Annotation of the Drosophila melanogaster euchromatic genome: a systematic review. *Genome biology* **3**, RESEARCH0083 (2002).
47 Slater, G. S. & Birney, E. Automated generation of heuristics for biological sequence comparison. *BMC bioinformatics* **6**, 31 (2005).
48 Suzek, B. E., Huang, H., McGarvey, P., Mazumder, R. & Wu, C. H. UniRef: comprehensive and non-redundant UniProt reference clusters. *Bioinformatics* **23**, 1282-1288 (2007).
49 She, R. *et al.* genBlastG: using BLAST searches to build homologous gene models. *Bioinformatics* **27**, 2141-2143 (2011).
50 Stanke, M., Schoffmann, O., Morgenstern, B. & Waack, S. Gene prediction in eukaryotes with a generalized hidden Markov model that uses hints from external sources. *BMC bioinformatics* **7**, 62 (2006).
51 Korf, I. Gene finding in novel genomes. *BMC bioinformatics* **5**, 59 (2004).
52 Besemer, J. & Borodovsky, M. GeneMark: web software for gene finding in prokaryotes, eukaryotes and viruses. *Nucleic acids research* **33**, W451-454 (2005).
53 Majoros, W. H., Pertea, M. & Salzberg, S. L. TigrScan and GlimmerHMM: two open source ab initio eukaryotic gene-finders. *Bioinformatics* **20**, 2878-2879 (2004).
54 Cantarel, B. L. *et al.* MAKER: an easy-to-use annotation pipeline designed for emerging model organism genomes. *Genome research* **18**, 188-196 (2008).

55 Liu, Q., Mackey, A. J., Roos, D. S. & Pereira, F. C. Evigan: a hidden variable model for integrating gene evidence for eukaryotic gene prediction. *Bioinformatics* **24**, 597-605, doi:10.1093/bioinformatics/btn004 (2008).
56 UniProt, C. Activities at the Universal Protein Resource (UniProt). *Nucleic acids research* **42**, D191-198 (2014).
57 Schnoes, A. M., Brown, S. D., Dodevski, I. & Babbitt, P. C. Annotation error in public databases: misannotation of molecular function in enzyme superfamilies. *PLoS computational biology* **5**, e1000605, doi:10.1371/journal.pcbi.1000605 (2009).
58 Jones, P. *et al.* InterProScan 5: genome-scale protein function classification. *Bioinformatics* **30**, 1236-1240 (2014).
59 Punta, M. *et al.* The Pfam protein families database. *Nucleic acids research* **40**, D290-301, doi:10.1093/nar/gkr1065 (2012).
60 Letunic, I., Doerks, T. & Bork, P. SMART 7: recent updates to the protein domain annotation resource. *Nucleic acids research* **40**, D302-305, doi:10.1093/nar/gkr931 (2012).
61 Mi, H., Muruganujan, A. & Thomas, P. D. PANTHER in 2013: modeling the evolution of gene function, and other gene attributes, in the context of phylogenetic trees. *Nucleic acids research* **41**, D377-386, doi:10.1093/nar/gks1118 (2013).
62 Pedruzzi, I. *et al.* HAMAP in 2013, new developments in the protein family classification and annotation system. *Nucleic acids research* **41**, D584-589, doi:10.1093/nar/gks1157 (2013).
63 Sigrist, C. J. *et al.* New and continuing developments at PROSITE. *Nucleic acids research* **41**, D344-347, doi:10.1093/nar/gks1067 (2013).
64 Wu, C. H. *et al.* PIRSF: family classification system at the Protein Information Resource. *Nucleic acids research* **32**, D112-114, doi:10.1093/nar/gkh097 (2004).
65 Haft, D. H. *et al.* TIGRFAMs and Genome Properties in 2013. *Nucleic acids research* **41**, D387-395, doi:10.1093/nar/gks1234 (2013).
66 Attwood, T. K. *et al.* The PRINTS database: a fine-grained protein sequence annotation and analysis resource--its status in 2012. *Database : the journal of biological databases and curation* **2012**, bas019, doi:10.1093/database/bas019 (2012).
67 Lupas, A., Van Dyke, M. & Stock, J. Predicting coiled coils from protein sequences. *Science* **252**, 1162-1164, doi:10.1126/science.252.5009.1162 (1991).
68 Krogh, A., Larsson, B., von Heijne, G. & Sonnhammer, E. L. Predicting transmembrane protein topology with a hidden Markov model: application to complete genomes. *Journal of molecular biology* **305**, 567-580, doi:10.1006/jmbi.2000.4315 (2001).
69 Kall, L., Krogh, A. & Sonnhammer, E. L. A combined transmembrane topology and signal peptide prediction method. *Journal of molecular biology* **338**, 1027-1036, doi:10.1016/j.jmb.2004.03.016 (2004).
70 Petersen, T. N., Brunak, S., von Heijne, G. & Nielsen, H. SignalP 4.0: discriminating signal peptides from transmembrane regions. *Nature methods* **8**, 785-786, doi:10.1038/nmeth.1701 (2011).
71 de Lima Morais, D. A. *et al.* SUPERFAMILY 1.75 including a domain-centric gene ontology method. *Nucleic acids research* **39**, D427-434, doi:10.1093/nar/gkq1130 (2011).

72 Lees, J. *et al.* Gene3D: a domain-based resource for comparative genomics, functional annotation and protein network analysis. *Nucleic acids research* **40**, D465-471, doi:10.1093/nar/gkr1181 (2012).
73 Kanehisa, M. Molecular network analysis of diseases and drugs in KEGG. *Methods in molecular biology* **939**, 263-275, doi:10.1007/978-1-62703-107-3_17 (2013).
74 Croft, D. *et al.* The Reactome pathway knowledgebase. *Nucleic acids research* **42**, D472-477, doi:10.1093/nar/gkt1102 (2014).
75 Morgat, A. *et al.* UniPathway: a resource for the exploration and annotation of metabolic pathways. *Nucleic acids research* **40**, D761-769, doi:10.1093/nar/gkr1023 (2012).
76 Caspi, R. *et al.* The MetaCyc database of metabolic pathways and enzymes and the BioCyc collection of Pathway/Genome Databases. *Nucleic acids research* **42**, D459-471, doi:10.1093/nar/gkt1103 (2014).
77 Li, L., Stoeckert, C. J., Jr. & Roos, D. S. OrthoMCL: identification of ortholog groups for eukaryotic genomes. *Genome research* **13**, 2178-2189 (2003).
78 Zhong, Y. F. & Holland, P. W. HomeoDB2: functional expansion of a comparative homeobox gene database for evolutionary developmental biology. *Evolution & development* **13**, 567-568 (2011).
79 Edgar, R. C. MUSCLE: multiple sequence alignment with high accuracy and high throughput. *Nucleic acids research* **32**, 1792-1797, doi:10.1093/nar/gkh340 (2004).
80 Stamatakis, A. RAxML version 8: a tool for phylogenetic analysis and post-analysis of large phylogenies. *Bioinformatics* **30**, 1312-1313, doi:10.1093/bioinformatics/btu033 (2014).
81 Katoh, K. & Standley, D. M. MAFFT multiple sequence alignment software version 7: improvements in performance and usability. *Molecular biology and evolution* **30**, 772-780, doi:10.1093/molbev/mst010 (2013).
82 Ferguson, L. *et al.* Ancient expansion of the hox cluster in lepidoptera generated four homeobox genes implicated in extra-embryonic tissue formation. *PLoS genetics* **10**, e1004698, doi:10.1371/journal.pgen.1004698 (2014).
83 Frickey, T. & Lupas, A. CLANS: a Java application for visualizing protein families based on pairwise similarity. *Bioinformatics* **20**, 3702-3704, doi:10.1093/bioinformatics/bth444 (2004).
84 Lartillot, N., Lepage, T. & Blanquart, S. PhyloBayes 3: a Bayesian software package for phylogenetic reconstruction and molecular dating. *Bioinformatics* **25**, 2286-2288, doi:10.1093/bioinformatics/btp368 (2009).
85 Lartillot, N. & Philippe, H. A Bayesian mixture model for across-site heterogeneities in the amino-acid replacement process. *Molecular biology and evolution* **21**, 1095-1109, doi:10.1093/molbev/msh112 (2004).
86 Gascuel, O. BIONJ: an improved version of the NJ algorithm based on a simple model of sequence data. *Molecular biology and evolution* **14**, 685-695 (1997).
87 Dereeper, A. *et al.* Phylogeny.fr: robust phylogenetic analysis for the non-specialist. *Nucleic acids research* **36**, W465-469, doi:10.1093/nar/gkn180 (2008).
